# LSD1 inhibition by tranylcypromine hydrochloride reduces alkali burn-induced corneal neovascularization and ferroptosis by suppressing HIF-1α pathway

**DOI:** 10.3389/fphar.2024.1411513

**Published:** 2024-07-26

**Authors:** Qian Deng, Yuelan Gao, Yujin Wang, Jiewen Mao, Yulin Yan, Zixian Yang, Yuyu Cong, Yanning Yang, Shanshan Wan

**Affiliations:** Department of Ophthalmology, Renmin Hospital of Wuhan University, Wuhan, Hubei, China

**Keywords:** alkali burn, corneal neovascularization, lysine-specific demethylase 1, tranylcypromine hydrochloride, HIF-1α, ferroptosis, angiogenesis, oxidative stress

## Abstract

**Background:**

Corneal neovascularization (CNV) is a sight-threatening condition that necessitates epigenetic control. The role of lysine-specific demethylase 1 (LSD1) in CNV remains unclear, despite its established significance in tumor angiogenesis regulation.

**Methods:**

An alkali burn-induced CNV mouse model was used *in vivo*. The effects of LSD1 inhibitor tranylcypromine hydrochloride (TCP) were examined through slit lamp, histological staining, and immunofluorescence. The expression of malondialdehyde (MDA), superoxide dismutase (SOD), and glutathione (GSH) levels were assessed in corneal tissues. Oxidative stress and ferrous ion expression during CNV were determined using 4-HNE, GPX4, and FerroOrange staining. *In vitro*, a hypoxia-reoxygenation (H/R) model was established using human umbilical vein endothelial cells (HUVECs) to study LSD1 or hypoxia-inducible factor (HIF-1α) knockdown and lentiviral overexpression of HIF-1α. The effects on HUVECs migration, invasion, and angiogenesis were evaluated through cell scratching assay, transwell migration assay and tube formation assay. The role of ferroptosis was investigated using ROS staining, FerroOrange staining, and key ferroptosis proteins. Further, The JAK2/STAT3 pathway’s involvement in CNV regulation was explored through in vivo experiments with subconjunctival injection of AG490.

**Results:**

The results showed a substantial correlation between corneal damage and LSD1 levels. In addition, HIF-1α expression was also elevated after alkali burns, and subconjunctival injection of TCP reduced corneal inflammation and neovascularization. Corneal alkali burns increased ROS levels and reduced antioxidative stress indicators, accompanied by elevated ferrous ion levels, which were reversed by TCP injection. In vitro, TCP or siRNAs inhibited H/R-induced ferroptosis and angiogenesis in HUVECs by affecting specific protein expressions and MDA, SOD, and GSH levels. HIF-1α levels, associated with ROS production, ferroptosis, and angiogenesis, increased during H/R, but were reversed by TCP or siRNA administration. HIF-1α overexpression counteracted the effects of LSD1 inhibition. Additionally, AG490 injection effectively reduced HIF-1α and VEGFA expression in the CNV model.

**Discussion:**

These findings suggest that LSD1 inhibition via the HIF-1α-driven pathway prevents angiogenesis, oxidative stress, and ferroptosis in corneal alkali burn-induced CNV, highlighting LSD1 as a potential therapeutic target.

## 1 Introduction

Angiogenesis is a fundamental biological process involved in age-related macular degeneration, diabetic retinopathy, and cancer. The cornea, a transparent tissue at the front of the eye, is crucial for vision and light refraction. In addition, the cornea serves as a mechanical barrier to protect the inside of the eye from harmful substances and microbes from the outside. Generally, the cornea is transparent and avascular and maintains its avascular state after treatment with antiangiogenic agents (Ambati et al., 2006). However, immunological diseases, chemical burns, trauma, and infection cause pathological alterations in the cornea that increase the expression of proangiogenic genes (Cursiefen et al., 2004). Corneal opacity is triggered by corneal neovascularization (CNV), which is characterized by the formation of new blood vessels from the corneal limbus toward its transparent core. Concomitantly, CNV-induced corneal edema impairs corneal transparency and adversely impacts visual acuity (Chang et al., 2001).

Through modulating transcriptional activity, epigenetic regulation—which comprises histone modifications, DNA methylation, and controls microRNA expression—plays essential roles in biological processes such as cell proliferation, death, inflammation, and neovascularization (Hogg et al., 2020; Das et al., 2021; Tan et al., 2021). Lysine-specific demethylase 1 (LSD1) regulates the late expression of downstream genes, which influences physiological activities. LSD1 removes monomethyl or dimethyl lysine nine from histone H3 (H3K9me1/2) (Shi et al., 2004; Lan et al., 2007). Prior research has shown that LSD1 is essential for oxygen–glucose deprivation/reoxygenation-induced pyroptosis in retinal ganglion cells and diabetic retinopathy (Zhao et al., 1979; Yu et al., 2022). Nevertheless, the function of LSD1 in CNV and the mechanism through which it works in this context remain unclear.

Ferroptosis is a unique form of programmed cell death that is linked to a variety of clinical events, such as liver injury, ischemia/reperfusion injury, neurological disorders, and cancer. Ferroptosis is characterized by an abnormal accumulation of lipid peroxides (Linkermann et al., 2014; Friedmann Angeli et al., 2019; Li et al., 2020; Mao et al., 2020) and has been implicated in ocular neovascularization (Wang et al., 2022). However, the precise processes through which ferroptosis functions in CNV remain unclear.

Excess reactive oxygen species (ROS) are produced when cells are subjected to damaging stimuli such as UV light and high metabolic activity (Hsueh et al., 2022). Superoxide anions (O^2−^), hydroxyl radicals (•OH), and peroxide radicals are examples of unstable free radicals with unpaired electrons that target a wide variety of cellular components, including DNA and proteins, leading to the breakdown of cell structure and loss of cell function. Numerous studies have demonstrated that oxidative stress plays a pivotal role in the pathogenesis of CNV (Wan et al., 2020; Wang et al., 2022). Notably, multiple disorders caused by oxidative stress have also been linked to LSD1 (Ma et al., 2021; Feng et al., 2022; Araki et al., 2023). However, how LSD1 modulates oxidative stress during CNV is unknown.

Hypoxia-inducible factor 1-alpha (HIF-1α) is a master regulator of the cellular response to hypoxia, orchestrating a transcriptional program that includes genes involved in angiogenesis and oxidative stress management. Under hypoxic conditions, HIF-1α accumulates and activates the transcription of various angiogenic factors, such as vascular endothelial growth factor (VEGF), which stimulates the proliferation and migration of endothelial cells to form new blood vessels. It has been demonstrated that downregulation of HIF-1α in HUVECs decreased the level of inflammation and oxidative stress in HUVECs (Zhao et al., 2021). The Janus kinase 2 (JAK2)/signal transducer and activator of transcription 3 (STAT3) pathway is a canonical signaling cascade that mediates diverse biological responses to extracellular cues, including cytokines and growth factors, and is known to be involved in the regulation of angiogenesis, inflammation, and cell survival. In gastric cancer (GC), activation of JAK2/STAT3 could promote HIF-1α/VEGFA axis, and bolsters macrophage polarization, thus facilitating GC metastasis and angiogenesis (Mu et al., 2021).

Given the significant regulatory roles of LSD1 and HIF-1α in oxidative stress and angiogenesis, it can be inferred that there may exist a mutual regulation between them. In this study, we examined the precise mechanism by which LSD1 regulates oxidative stress in CNV post-alkali burn and further examined its underlying relationship with ferroptosis in both vivo and vitro model. Our study elucidates the role of LSD1 in regulating oxidative stress and ferroptosis via the HIF-1α pathway, consequently facilitating the progression of CNV. These findings contribute significant insights to the clinical strategies for CNV treatment and can serve as a reference for potential therapeutic interventions.

## 2 Materials and methods

### 2.1 Antibodies and reagents

The LSD1 inhibitor, tranylcypromine hydrochloride (TCP) and the JAK2/STAT3 inhibitor AG490 were acquired from MedChemExpress Biotechnology (United States). According to the manufacturer’s instructions, both inhibitors were dissolved in dimethyl sulfoxide (DMSO). SiRNAs and polymerase chain reaction (PCR) primers were obtained from Sangon Biotech. The antibodies utilized in the study were obtained from various sources: LSD1 from Abcam; Phospho-Stat3 (Tyr705) (p-STAT3), STAT3, Histone H3 from Cell Signaling Technology; platelet/endothelial cell adhesion molecule 1 (CD31) from Invitrogen; vascular endothelial growth factor A (VEGFA), β-actin, GPX4, HMOX1, ACSL4, SLC7A11from Proteintech (Wuhan, China); JAK2, Phospho-JAK2 (Tyr931), HIF-1α, Di-Methyl-Histone H3 (Lys9) (H3K9me2) from Affinity (Jiangsu, China); Fe^2+^ Indicator Probes was purchased from DOJINDO (Japan). To determine the oxidative status, malondialdehyde (MDA), superoxide dismutase (SOD), Glutathione (GSH) assay kits were purchased from Beyotime (Shanghai, China). More detailed information such as the catalog number are listed in the Supplementary Material.

### 2.2 Mouse model of alkali-induced CNV

The Experimental Animal Centre of Wuhan University’s Medical College (Wuhan, China) supplied male C57BL/6 mice that were 6–8 weeks old and weighed 20–25 g. The Guidelines for the Care and Use of Laboratory Animals were followed throughout (approval number: No.20220504A). The Wuhan University Experimental Animal Committee approved all of the animal experiments. The mice were allowed free access to water and standard chow, and their environment was maintained between 20°C and 22°C with a 12-hour light/dark cycle.

An alkali burn (AB) approach was used to generate CNV as previously described. All mice were anesthetized with an intraperitoneal injection of pentobarbital (50 mg/kg). The central cornea of the right eye was covered for 30 sec with a filtered paper disc (diameter of 2.5 mm) soaked in sterile sodium hydroxide (1 M). The cornea was then promptly rinsed with 0.9% saline solution for 1 min. Following alkali burn, the animals received subconjunctival injections of DMSO or various dosages of the TCP once a day for 3, 7, or 10 days after the alkali burn. The experiment was terminated by euthanasia through cervical dislocation, The eyeballs were then harvested, and the corneas were isolated for subsequent experimentation.

Mice were randomly assigned to different groups based on specific experiment demand: prior to modeling (control group, n = 20), 3 days post-modeling (3d group, n = 20), 7 days post-modeling (7d group, n = 20), and 10 days post-modeling (10d group, n = 20). This categorization aids in assessing the inducive effect of NaOH on CNV and in determining the most appropriate experimental time points. Subsequently, experimental time points were selected based on the expression levels of LSD1. For the purpose of screening the optimal concentration of TCP, mice were further divided into different groups: control group (no modeling, n = 20), AB + DMSO group (treated with DMSO solution, n = 20), AB + T1 group (treated with 1 mg/kg TCP, n = 20), AB + T5 group (treated with 5 mg/kg TCP, n = 20), and AB + T10 group (treated with 10 mg/kg TCP, n = 20). To further investigate the regulatory effects of LSD1 on the JAK2/STAT3/HIF-1α pathway, mice were divided into control group (n = 20), AB + DMSO group (n = 20), AB + TCP group (n = 20), and AB + AG490 group (n = 20). At specified time points, mice were anesthetized, and eyeballs were harvested, marking the end of the experiment for subsequent analyses. Images were obtained using a slit lamp microscopy 10-days after alkali burn. All experiments were repeated three times to ensure the reliability and consistency of the results.

### 2.3 Cell culture and treatment

Acute oxidative stress is an upstream regulator of inflammation and CNV formation in corneal injury caused by alkali burns (Gu et al., 2016). Our previous research highlighted the important role of hypoxia and oxidative stress in CNV pathogenesis (Wan et al., 2020). Therefore, in this study, we use hypoxia/reoxygenation (H/R) induced *in vitro* model to simulate the hypoxia conditions on the development of CNV.

Human umbilical vein endothelial cells (HUVECs) purchased from the American Type Culture Collection (Manassas, VA, United States) were cultured at 37°C in Dulbecco’s modified Eagle’s medium (DMEM) supplemented with 10% fetal bovine serum (FBS), 100 units/mL penicillin, and 100 μg/mL streptomycin in an atmosphere containing 5% carbon dioxide (CO_2_) and 95% air. The following describes how the cell hypoxia/reoxygenation (H/R) model was developed. HUVECs were subjected to hypoxic conditions (37°C, 1% oxygen, 94% nitrogen, and 5% CO_2_) for 12 h in medium without glucose or serum in a tri-gas incubator (Thermo Scientific™ Forma™ II series Water jacket CO_2_ incubator, Waltham, United States). The cells were subsequently cultivated under standard conditions (5% CO_2_) and reoxygenated for 6, 12, and 24 h after the medium was changed, following the experimental design. The control group was cultivated under standard conditions, which included 95% air and 5% CO_2_.

### 2.4 Quantitative real-time PCR (qPCR)

Following the manufacturer’s instructions, total RNA was extracted from frozen corneal tissues and HUVECs using the RNA Isolater Total RNA Extraction Reagent (R401-01, Vazyme, Nanjing, China). HiScript II Q RT SuperMix for qPCR (R222-01, Vazyme, Nanjing, China) was then utilized to reverse transcribe the resulting cDNA. β-actin expression was used as an internal reference for all PCR assays. 2X Universal SYBR Green Fast qPCR Mix (RK21203, ABclonal, Wuhan, China) was used for the qRT‒PCR study. Sangon Biotech generated the qRT‒PCR primers for the target genes (shown below).

LSD1: 5′- GGT​GAG​CTC​TTC​CTC​TTC​TGG-3’ (F)

5′- TCGGCCAACAATCACATCGT-3′(R)

IL-1β: 5′- GAA​ATG​CCA​CCT​TTT​GAC​AGT​G-3’ (F)

5′-TGG​ATG​CTC​TCA​TCA​GGA​CAG-3’ (R)

IL-6: 5′- CTG​CAA​GAG​ACT​TCC​ATC​CAG-3’ (F)

5′- AGTGGTATAGACAGGTCTGTTGG-3′(R)

TNF-α: 5′-CAG​GCG​GTG​CCT​ATG​TCT​C-3’ (F)

5′- CGA​TCA​CCC​CGA​AGT​TCA​GTA​G-3’ (R)

β-Actin: 5′-GAA​ATC​GTG​CGT​GAC​ATC​AAA-3’ (F)

5′- TGTAGTTTCATGGATGCCACAG-3′(R)

Routine qRT-PCR was performed as follows: 95°C for 3 min, followed by 40 cycles at 95°C for 5 sec, 60°C for 30 sec, and 72°C for 1 min. Relative gene expression levels were calculated with the 2^−ΔΔCT^ method (Livak and Schmittgen, 2001).

### 2.5 Western blot

Total protein was isolated from corneal tissues and HUVECs using a commercial kit (R0018, Beyotime, Shanghai, China). For quantification, a protein assay kit (P0010, Beyotime, Shanghai, China) was used following the manufacturer’s instructions. Proteins (40 µg/lane) were separated by electrophoresis on 10% sodium dodecyl sulfate‒polyacrylamide gels and then transferred to polyvinylidene fluoride membranes. Five percent fat-free milk was used to block the membranes for 2 h at room temperature. The membranes were then incubated with the following primary antibodies at specific dilutions: LSD1 (1:2000), β-actin (1:12000), VEGFA (1:2000), p-JAK2 (1:500), JAK2 (1:500), p-STAT3 (1:1000), STAT3 (1:1000), H3 (1:2000), H3K9me2 (1:2000), HIF-1α (1:500), GPX4 (1:2000), HMOX1 (1:2000), ACSL4 (1:1000), and SLC7A11 (1:2000). Tween 20 buffer and Tris-buffered saline were utilized to remove extra primary antibodies from the membranes. The membranes were incubated with a suitable secondary antibody (1:5000) for 2 h at 37°C. Excess secondary antibody was removed, and enhanced chemiluminescence was used to detect the signals of the detected proteins. The protein concentrations were examined using ImageJ Software (National Institutes of Health, Bethesda, MD, United States), and the results are shown relative to the protein concentrations observed in the control group, and were further compared to the internal control protein β-actin for quantitative analysis.

### 2.6 Evaluation of corneal opacity and neovascularization

Utilization of slit-lamp microscopy facilitated the assessment of corneal opacity and neovascularization at designated time points post-model induction, specifically on days 0, 3, 7, and 10. Quantitative analysis and grading of the extent of CNV and edema were conducted based on captured corneal images. The area affected by CNV was computed via the formula:

A = (C/12) × 3.14 × [r^2^ − (r − l)^2^], where r is the corneal radius, l is the length of the blood vessels, and C denotes the number of clock hours along the corneal circumference exhibiting neovascularization. Corneal edema, serving as an indicator of the inflammatory status, was predominantly evaluated using a scoring protocol designed for the measurement of corneal opacity (Bai et al., 2016).

### 2.7 Hematoxylin and eosin staining

To assess the inflammatory response of the cornea after alkali burns, hematoxylin-eosin staining was applied to evaluate the number of corneal inflammatory cells as well as the thickness of the corneal stroma. A minimum of 5 murine eyes per group were included, with corneas harvested for the following procedures. Corneal tissue sections (7 µm thick) were fixed with 4% paraformaldehyde and embedded in paraffin and then subjected to hematoxylin-eosin staining. Inflammatory cell counts were conducted on the maximum cross-sections of the cornea, ensuring no fewer than three sections per paraffin-embedded corneal block were selected for this analysis.

### 2.8 Immunohistochemistry

Immunohistochemical staining was performed using a commercial kit (PV-6002, ZSGB, Beijing, China). Briefly, corneal tissues were fixed in 4% paraformaldehyde, subsequently embedded in paraffin, and sectioned into slices of 7 μm in thickness. Endogenous enzymatic activity was quenched utilizing 3% hydrogen peroxide, succeeded by antigen retrieval facilitated by microwave-induced heating. Post-blocking with serum, the tissue sections underwent overnight incubation at 4°C with a primary antibody specific for H3K9me2 (diluted 1:50), GPX4 (diluted 1:100) and 4-HNE (diluted 1:100). Subsequently, the sections were exposed to secondary antibodies at ambient temperature for a duration of 1 h and subsequently developed using 3,3′-diaminobenzidine (DAB). A minimum of 4 murine eyes per group were included, and five distinct fields were chosen randomly and a microscope was used to assess the staining intensity. Positive staining cells were quantified using ImageJ software.

### 2.9 Immunofluorescence

Corneal tissues were fixed, dehydrated, cleared, and embedded in paraffin before being sectioned and mounted on glass slides. After deparaffinization and antigen retrieval, sections were permeabilized and blocked to prevent non-specific binding. For HUVECs, they were fixed with 4% paraformaldehyde, place onto a Petri dish with a glass coverslip, and incubated on ice for 20 min. After 10 min of incubation in 0.5% Triton X-100, the cells were blocked for 30 min with 1% bovine serum albumin. Tissues were then incubated with primary antibodies overnight, followed by washing and incubation with fluorophore-conjugated secondary antibodies. Finally, sections were counterstained with DAPI, mounted with an anti-fade medium, and visualized using a fluorescence microscopy (Olympus, BX53, Japan). Non-specific staining was assessed with control sections processed in parallel.

### 2.10 Measurement of ROS production

The levels of ROS were quantified using a ROS Assay Kit (S0033S, Beyotime, Shanghai, China). HUVECs were incubated with medium containing 20 µM dichlorodihydrofluorescein diacetate solution for 30 min at 37°C in the dark. The cells were then rinsed with serum-free cell culture media. An inverted fluorescence microscope (Olympus, IX71, Japan) was used to measure the staining that reflected the ROS levels. Controls were included to ensure the specificity and sensitivity of the ROS detection, with all procedures carried out to minimize light exposure and prevent photooxidation.

### 2.11 Tube formation assay

After cultivation and reaching optimal confluency, HUVECs were harvested and seeded onto Matrigel (354234, Corning, NY, United States) coated 96-well plates at a density conducive to the formation of capillary-like structures. Following seeding, HUVECs were subjected to hypoxia conditions as previously described for 12 h, and were subsequently cultivated under standard conditions and reoxygenated for 24 h. Periodic monitoring was performed to assess the degree of tubular structure formation. After 24 h of hypoxia/reoxygenation (H/R) treatment, and following the application of TCP and siRNA, an inverted microscope (Olympus, BX53, Japan) was employed to qualitatively evaluate the capillary-like tube creation, and photographs were taken for subsequent quantitative analysis of the tube lengths and branching points, indicative of the angiogenic potential of the cells under the experimental conditions.

### 2.12 Cell scratching assay

Initially, HUVECs (1 × 10^5^ cells/well) were seeded into six-well plates. HUVECs were cultured under standard conditions. After the cells reached 80% confluence, the culture media were subjected to 1% serum starvation for 12 h. Once the cells had completely grown, a 200 μL pipette tip was used to form a scratch (Gao et al., 2024). Then, five groups were created for the experiment: control, H/R, H/R + TCP (50 ng/mL), and H/R + si-LSD1. The appropriate chemicals were added after wound formation, and images were obtained immediately and 24 h later.

### 2.13 Transwell migration assay

For each Transwell migration assay, the upper chamber was flooded with complete media supplemented with or without the LSD1 inhibitor under normal or H/R conditions after the HUVECs (5 × 10^4^ cells) were starved overnight. The lower chamber was filled with 500 μL of DMEM medium supplemented with 10% fetal bovine serum. After 24 h, the cells were immersed in 3% paraformaldehyde for 15 min. The cells were then subjected to crystal violet staining and were counted via optical microscopy (Olympus, BX53, Japan).

### 2.14 Cell viability

HUVECs were seeded onto 96-well plates at a density of 2 × 10^3^ cells per well and allowed to adhere overnight. Subsequently, the cells were segregated into distinct groups to undergo H/R modeling, and were subjected to various TCP concentrations (0, 1, 2.5, 5, 10 μM) for treatment. Control group and DMSO group were set up for comparison. Cell viability was assessed by measuring the absorbance at 450 nm using a microplate reader (Thermo Fisher Scientific, Waltham, United States) and a Cell Counting Kit-8 assay kit (BS350A, Biosharp, Hefei, China).

### 2.15 Lentiviral infection

HUVECs were transduced with a lentiviral vector expressing HIF-1α. Initially, HUVECs were cultured in complete medium until they reached 40%–50% confluence. Subsequently, the cells were incubated with the HIF-1α overexpressing lentivirus (multiplicity of infection, MOI = 30) in the presence of 4 μg/mL polybrene to enhance viral entry and ensure effective transduction. After 24 h of incubation, the medium containing the virus was replaced with fresh complete growth medium to minimize cytotoxic effects. The transduction efficiency was assessed 72 h post-transduction using Western blot analysis for HIF-1α expression.

### 2.16 Small interfering RNA (siRNA) transfection

Plating was performed on the basis of HUVECs cell density so that the optimal cell density on the day of transfection was between 80% and 90%. Lipofectamine 3000 reagent (Thermo Fisher Scientific, Waltham, United States) containing siRNA (100 µM) (Sangon Biotech, China) was used for transfection. 48 h after transfection, the cells were subjected to hypoxia/reoxygenation conditions. WB and qRT‒PCR assays were used to determine the effects of siRNA transfection on protein and mRNA expression.

### 2.17 Measurement of malondialdehyde (MDA), superoxide dismutase (SOD), and glutathione (GSH) levels

The MDA, SOD, and GSH activities in corneal tissues and HUVECs were measured using kits acquired from Beyotime, China. MDA is widely recognized as a biomarker for lipid peroxidation. It reacts with thiobarbituric acid (TBA) under high temperature and acidic conditions to form a red MDA-TBA adduct that exhibits peak absorption at 535 nm, facilitating its quantification through spectrophotometry (Bao et al., 2021). SOD serves as a critical antioxidant enzyme in biological systems. It inhibits the reaction between superoxide anions (O_2_^−^), generated catalytically by xanthine oxidase (XO), and WST-8. The inhibition of this reaction by SOD can be quantified by colorimetric analysis of the WST-8 product to determine the enzymatic activity of SOD (Weng et al., 2021). Glutathione reductase reduces oxidized glutathione (GSSG) to its reduced form (GSH), which then reacts with the chromogenic substrate DTNB to produce the yellow compound TNB and GSSG. Measurement at an absorbance of 412 nm allows for the quantification of total glutathione levels (Chen et al., 2010).

Measurements of SOD, MDA, and GSH were performed using the blank group as a control, per the manufacturer’s instructions. Briefly, corneal tissues were homogenized or HUVEC cells lysed in accordance with the instructions provided by the kit. Following centrifugation, the supernatant was collected for the preparation of requisite working solutions as well as to delineate the standard curves necessary for subsequent quantification. Finally, the content was determined by measuring the absorbance in a 96-well plate using a microplate reader (Thermo Fisher Scientific, Waltham, United States) at specific wavelengths.

### 2.18 Detection of Fe^2+^ in HUVECs

The Fe^2+^ concentration was measured using a FerroOrange probe (F374, DOJINDO, Japan), a highly sensitive fluorescent dye designed specifically for the detection of ferrous ions. HUVECs were cultured under standard conditions until reaching optimal confluency (70%–80%) and washed with Hank’s Balanced Salt Solution (HBSS) for three times. The medium was then replaced with fresh medium containing TCP and incubated further at 37°C, 5% CO_2_. Following this, the cells were washed again three times with HBSS. A working solution of FerroOrange at a concentration of 1 µM was added to the cells, which were then incubated for 30 min while shielded from light. A fluorescence microscope (Olympus IX71, Japan) was used to obtain images.

### 2.19 Statistical analysis

GraphPad Prism version 5.0 (GraphPad Software, La Jolla, CA, United States) was used for the statistical analysis. All of the data are presented as means ± standard errors of the means. For comparisons among multiple groups, analysis of variance (ANOVA) was utilized, whereas for comparisons between two groups, the Student’s two-tailed *t*-test was employed. Statistical significance was indicated by a *p*-value < 0.05.

## 3 Results

### 3.1 Alkali burn induced corneal inflammation and neovascularization in mice

**FIGURE 1:**
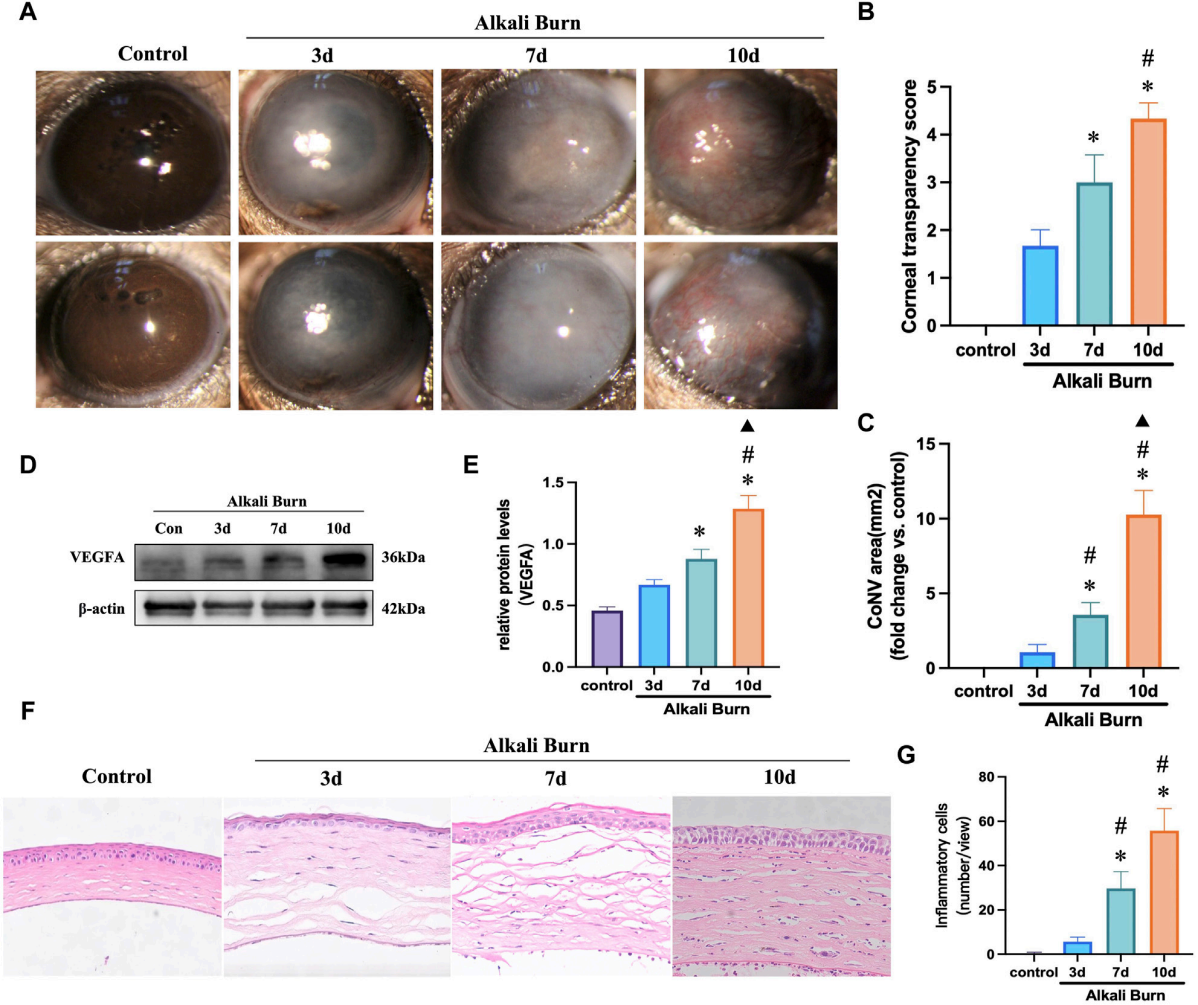
Corneal inflammation and neovascularization induced by alkali burn. **(A–C)** Corneal neovascularization and transparency. **(D, E)** VEGFA protein concentrations were measured on various days postburn. **(F, G)** Hematoxylin-eosin (HE) staining were utilized to delineate the temporal progression of corneal inflammation following alkali burn across various time points, with a concurrent quantitative assessment of inflammatory cell infiltration (200×; scale bars = 50 μm) (n = 8),*p < 0.05 versus the control group, #*p* < 0.05 versus 3 days group, ▲*p* < 0.05 versus 7 days group.

Three days post-model induction, slit-lamp microscopy revealed an onset of acute inflammatory response accompanied by corneal stroma edema, and the presence of sparse corneal neovascularization was noted. By the seventh day, there was an exacerbation of the inflammatory process, a discernible reduction in corneal clarity, and the vasculature within the keratitis-afflicted murine model displayed signs of congestion and dilation. On the 10th day, a marked decrease in corneal transparency was observed, along with a central progression of corneal neovascularization characterized by the formation of extensive anastomosing branches. Quantitative analysis demonstrated significant variances in corneal stroma opacity scores and the extent of neovascularization between the control cohort and the group evaluated at day 10 (*p* < 0.05; Figures 1A–C). Concurrently, the expression levels of VEGFA were significantly elevated on day 10 (Figures 1D, E). Histopathological examination conducted on day 3 post-injury displayed mild corneal stromal edema and minimal infiltration by inflammatory cells. Progressing to day seven, stromal matrix edema was intensified, and there was an upsurge in inflammatory cell presence. By the 10th day, both epithelial and stromal edema peaked, and there was a substantial increase in the infiltration of inflammatory cells (Figures 1F, G).

The experimental findings confirm the successful establishment of a CNV model precipitated by an alkali burn. In light of these observations, the pronounced neovascularization noted at day 10 post-injury was identified as an optimal time point for subsequent investigations, herein referred to as the alkali burn group.

### 3.2 LSD1 was upregulated after corneal alkali burn injury

**FIGURE 2:**
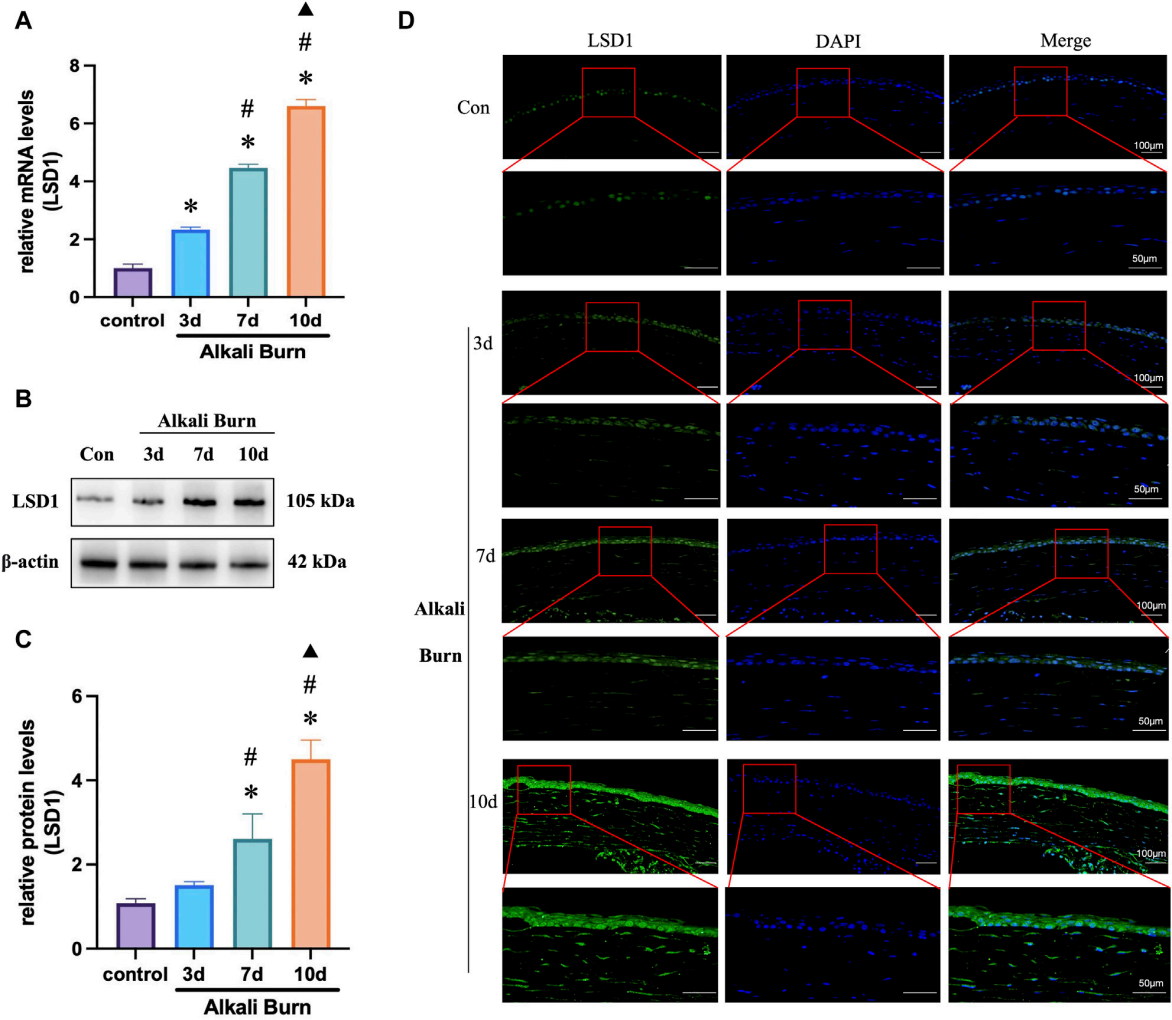
LSD1 expression was increased in corneas subjected to alkali burn injury. **(A)** RT‒qPCR was used to measure the levels of LSD1 at 3, 7, and 10 days postburn and expression levels were normalized to that observed in the control group. In **(B, C)**, LSD1 protein concentrations were measured on various days postburn, and the fold change in LSD1 expression was calculated by comparison to that in the control group. **(D)** Images of LSD1 immunofluorescence in ocular tissues 3, 7, and 10 days following alkali burn injury. n = 6, the data are expressed as the mean ± SEM (400×; scale bars = 50 μm). *p < 0.05 versus the control group, #*p* < 0.05 versus 3 days group, ▲*p* < 0.05 versus 7 days group.

LSD1 expression was first measured by WB (Figures 2B, C) and PCR (Figure 2A) 3, 7, and 10 days after alkali burn injury. LSD1 expression increased significantly with increasing postburn duration, which was especially evident 10 days after alkali burn. Furthermore, immunofluorescence examination revealed that LSD1 was primarily located in the nucleus and that its expression was greater on day 10 following alkali burn (Figure 2D). These findings showed that LSD1 may play a role in damage caused by alkali burns; therefore, all following experiments were conducted at 10 days postburn.

### 3.3 LSD1 inhibition reduced alkali burn-induced corneal damage

**FIGURE 3:**
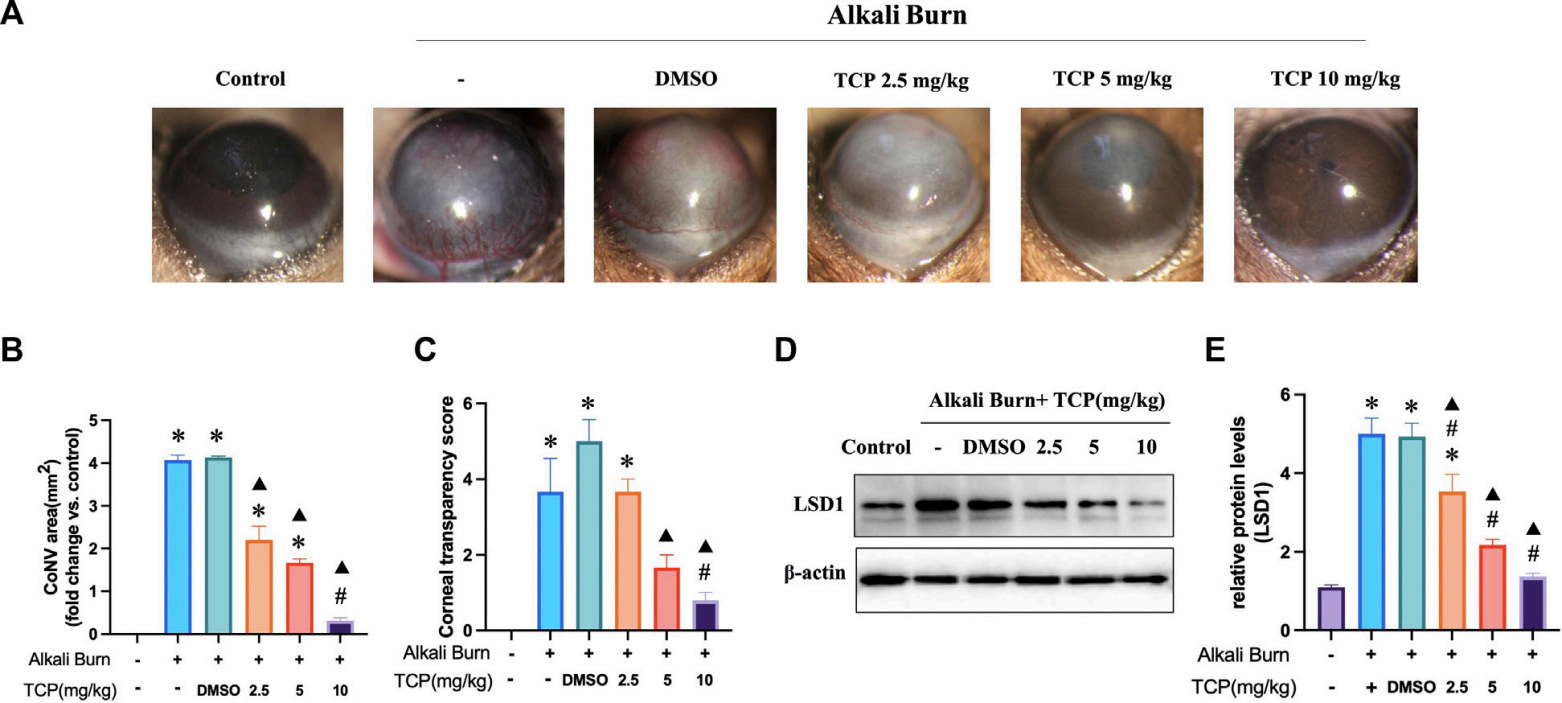
Tranylcypromine hydrochloride (TCP) treatment for alkali burn-induced corneal damage. **(A)** Using a slit lamp, microscopy images of corneal neovascularization induced by alkali burns were obtained on day 10. **(B, C)** Quantitative evaluation of the neovascularization region and corneal transparency score. LSD1 expression in mice treated with TCP at doses of 2.5, 5, and 10 mg/kg is shown in **(D, E)**, along with the fold change in LSD1 expression as determined by comparison with LSD1 expression in the control group. n = 6, the data are expressed as the mean ± SEM. *p < 0.05 versus the control group, #*p* < 0.05 versus alkali burn group, ▲*p* < 0.05 versus alkali burn + DMSO group.

Tranylcypromine hydrochloride (TCP) was used to suppress LSD1. Based on the morphological data obtained with a slit lamp, we obtained representative images of the cornea on day 10 after alkali burn. These images revealed significant pathological vascular proliferation and corneal opacity in the corneas subjected to alkali burns, which was reversed by subconjunctival injection of TCP (Figures 3A–C). The WB results demonstrated that TCP administration inhibited LSD1 expression, with the most significant suppression occurring at a dose of 10 mg/kg (Figures 3D, E). These findings imply that corneal damage caused by alkali burns is significantly reduced when LSD1 expression is inhibited.

### 3.4 Inhibition of LSD1 effectively reduces VEGFA expression and inflammation in alkali-burned corneas

**FIGURE 4:**
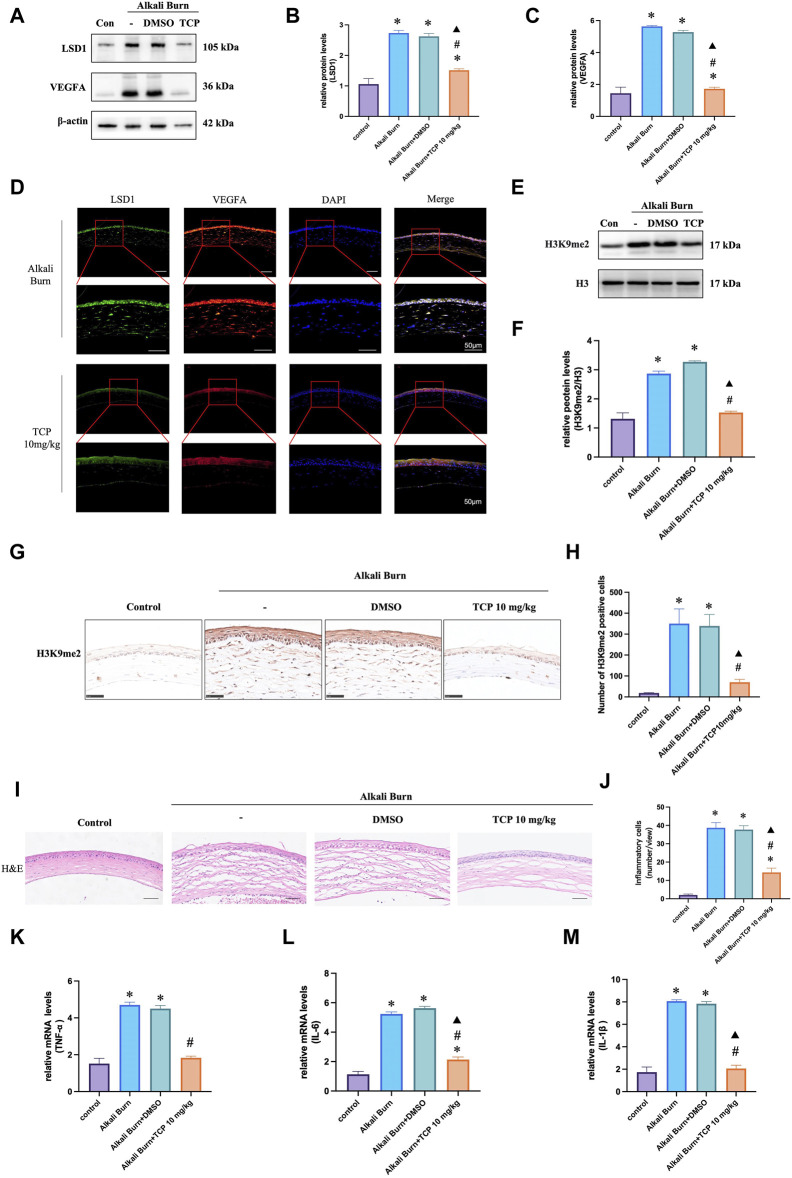
VEGFA expression in corneas 10 days after alkali burn injury was decreased by TCP treatment. Western blotting was used to measure LSD1 and VEGFA expression in **(A–C)**, and the fold change in expression was compared to that in the control group. **(D)** Double immunofluorescence analysis of VEGFA and LSD1 in corneas following alkali burn injury (10 days) (400×; scale bars = 50 μm). **(E, F)** show H3K9me2 expression as determined by Western blot analysis and the fold change in expression compared to that in the control group. **(G, H)** Immunohistochemistry for H3K9me2 in the corneas of mice subjected to alkali burn injury with or without TCP treatment. (400×; scale bars = 50 μm). **(I, J)** Following alkali burn injury, inflammatory cell infiltration was detected by H&E staining (400×; scale bars = 50 μm). **(K–M)** Following alkali burn injury qRT‒PCR was used to detect the mRNA levels of TNF-α and IL-1β, which are proinflammatory cytokines. n = 6, the data are expressed as the mean ± SEM. *p < 0.05 versus the control group, #*p* < 0.05 versus alkali burn group, ▲*p* < 0.05 versus alkali burn + DMSO group.

The WB results demonstrated that after subconjunctival TCP injection, the expression of H3K9me2 steadily decreased, and that the expression of VEGFA decreased with increasing TCP concentration. This finding was confirmed by immunohistochemistry and immunofluorescence analyses (Figures 4A–H). Furthermore, HE (Figures 4I, J) and qRT‒PCR (Figures 4K‒M) analyses following TCP injection revealed a significant decrease in the level of inflammation in alkali-burned corneas.

### 3.5 LSD1 inhibition reduced alkali burn-induced ferroptosis and oxidative stress

**FIGURE 5:**
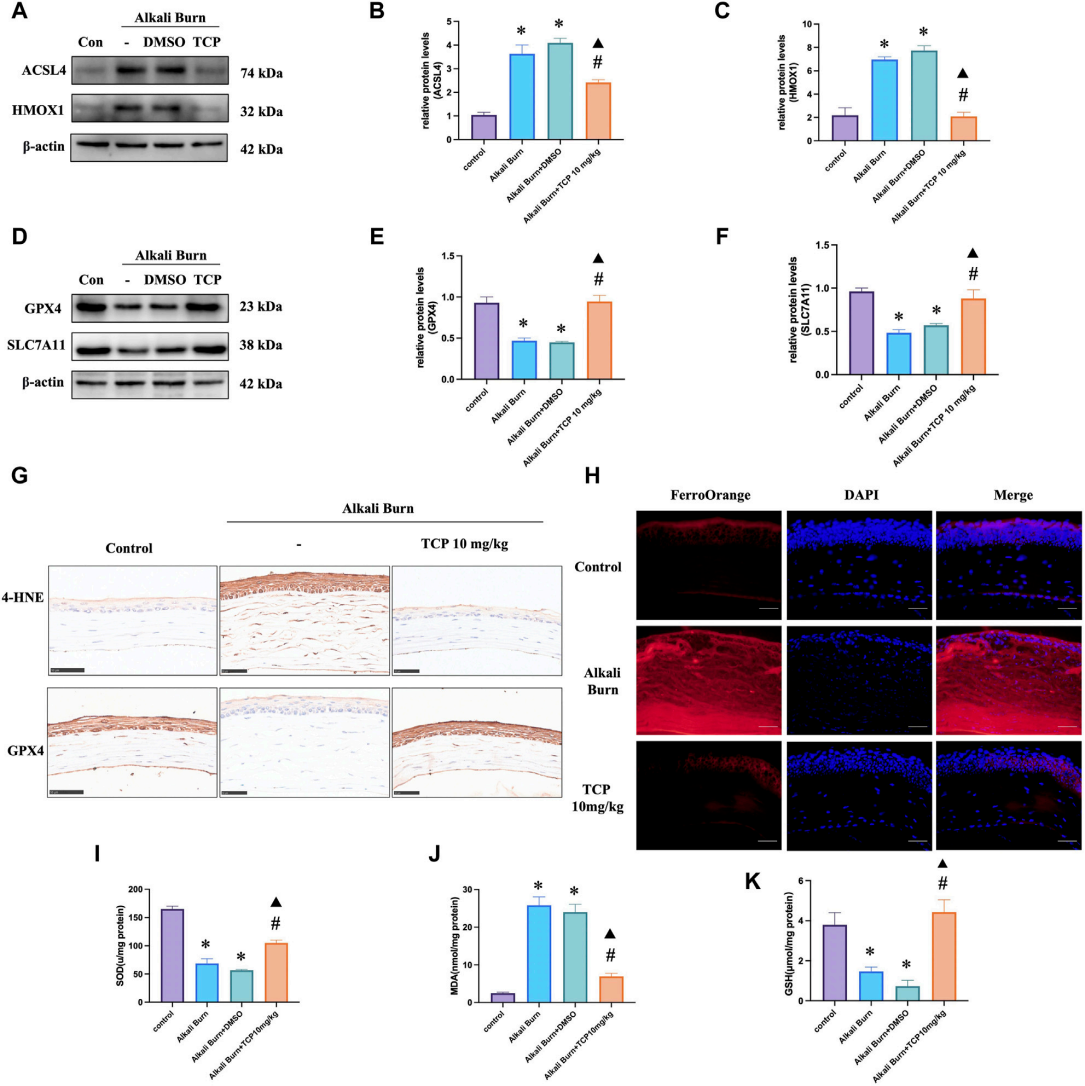
Ferroptosis and oxidative stress caused by alkali burn observed in the cornea 10 days after injury were decreased by tranylcypromine hydrochloride (TCP) treatment. **(A–F)** TCP modulation of ACSL4, HMOX1, GPX4 and SLC7A11 expression following corneal alkali burn injury. The fold change in expression was determined by comparison to the expression measured in the control group. **(G)** TCP-mediated regulation of GPX4 and 4-HNE levels in mice after corneal alkali burn injury (400×; scale bars = 50 μm). **(H)** The effect of TCP regulation on tissue Fe^2+^ levels in mice after corneal alkali burn injury (400×; scale bars = 50 μm). **(I–K)** The effect of TCP administration on SOD activity, GSH levels, and MDA levels in mice subjected to ocular alkali burn injury. n = 6, the data are expressed as the mean ± SEM. *p < 0.05 versus the control group, #*p* < 0.05 versus alkali burn group, ▲*p* < 0.05 versus alkali burn + DMSO group.

We conducted the following investigations to understand how TCP affects ferroptosis and oxidative stress *in vivo*. The findings demonstrated that there was an increase in the expression of ACSL4, HMOX1 (Figures 5A–C), MDA (Figure 5J), and 4-HNE (Figure 5G) following corneal alkali burn injury. TCP administration reversed these changes. Following alkali burn, tissue Fe^2+^ staining significantly increased in both intensity and range, and after receiving TCP therapy, it diminished (Figure 5H). However, following corneal alkali burn injury, GPX4 and SLC7A11 expression (Figures 5D–F), as well as SOD activity (Figure 5I) and GSH levels (Figure 5K), were reduced; moreover, TCP therapy reversed these changes.

### 3.6 LSD1 and VEGFA expression was elevated after H/R in HUVECs

**FIGURE 6:**
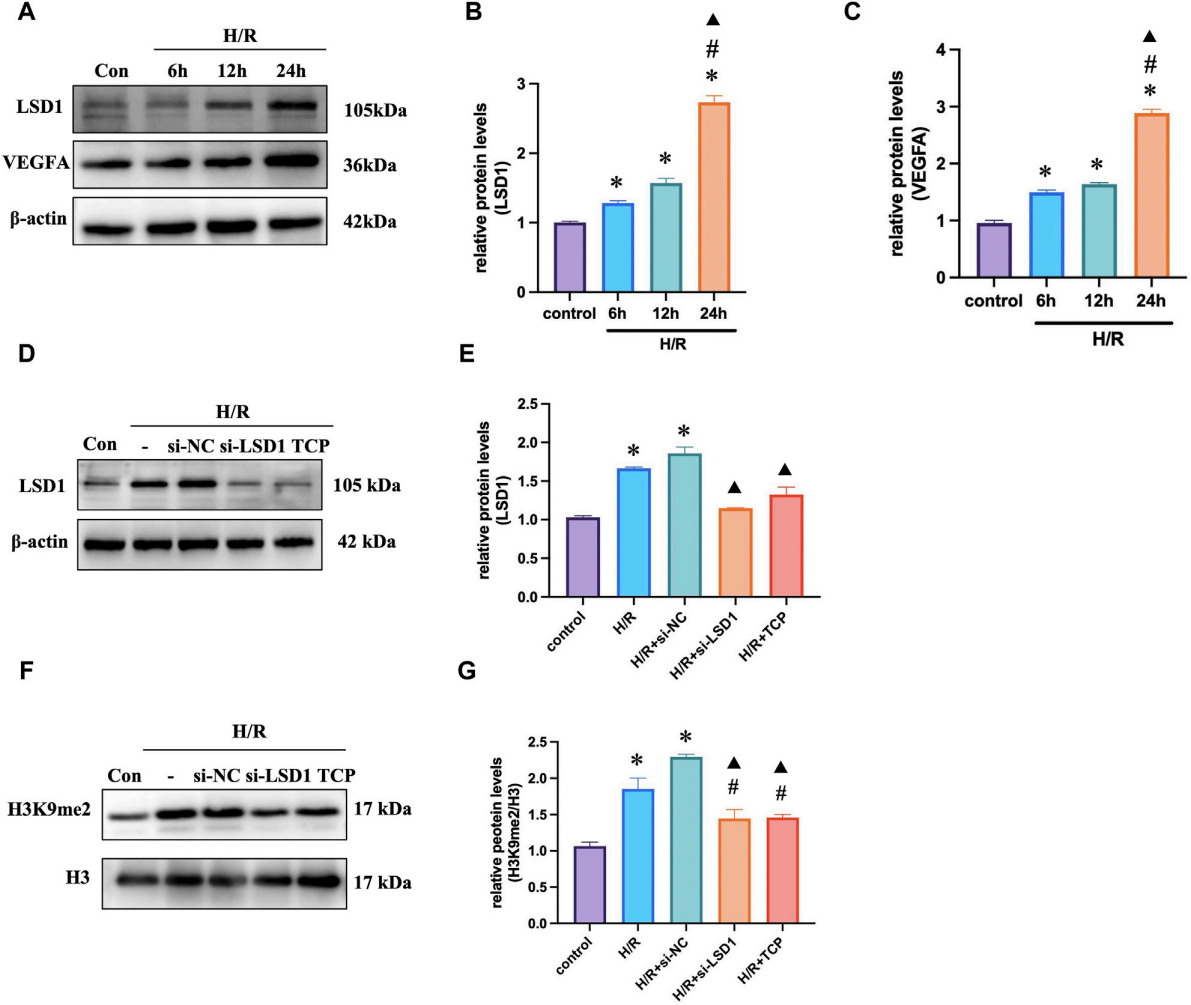
LSD1 expression increased *in vitro* during H/R. **(A–C)** Quantification of the changes in LSD1 and VEGFA expression compared to that in the control group and Western blot analysis of LSD1 and VEGFA expression after cells were reoxygenated for various times. **(D–G)** After inhibiting LSD1 expression with TCP or siRNA targeting LSD1, LSD1 expression and H3K9me2 levels were analyzed using Western blotting. For n = 6, the data are expressed as the mean ± SEM. For **(A–C)**, *p < 0.05 versus the control group (no treatment group), #*p* < 0.05 versus H/R 6 h group, ▲*p* < 0.05 versus H/R 12 h group. For **(D–G)**, *p < 0.05 versus the control group, #*p* < 0.05 versus H/R group, ▲*p* < 0.05 versus H/R + si-NC group.

Initially, we investigated the potential effects of various reoxygenation periods on LSD1 and VEGFA expression. We discovered that the expression of these genes was considerably greater in the H/R group than in the control group, and this difference was more noticeable after 24 h of reoxygenation (Figures 6A–C). The LSD1 gene was silenced *in vitro* using short interfering RNA (siRNA). The LSD1 protein levels were considerably reduced by TCP treatment or gene knockdown (Figures 6D, E). Furthermore, Figures 5F, G shows that the H3K9me2 level correlated with LSD1 expression.

### 3.7 LSD1 inhibition reduced H/R-induced angiogenesis, ferroptosis, and oxidative stress in HUVECs

**FIGURE 7:**
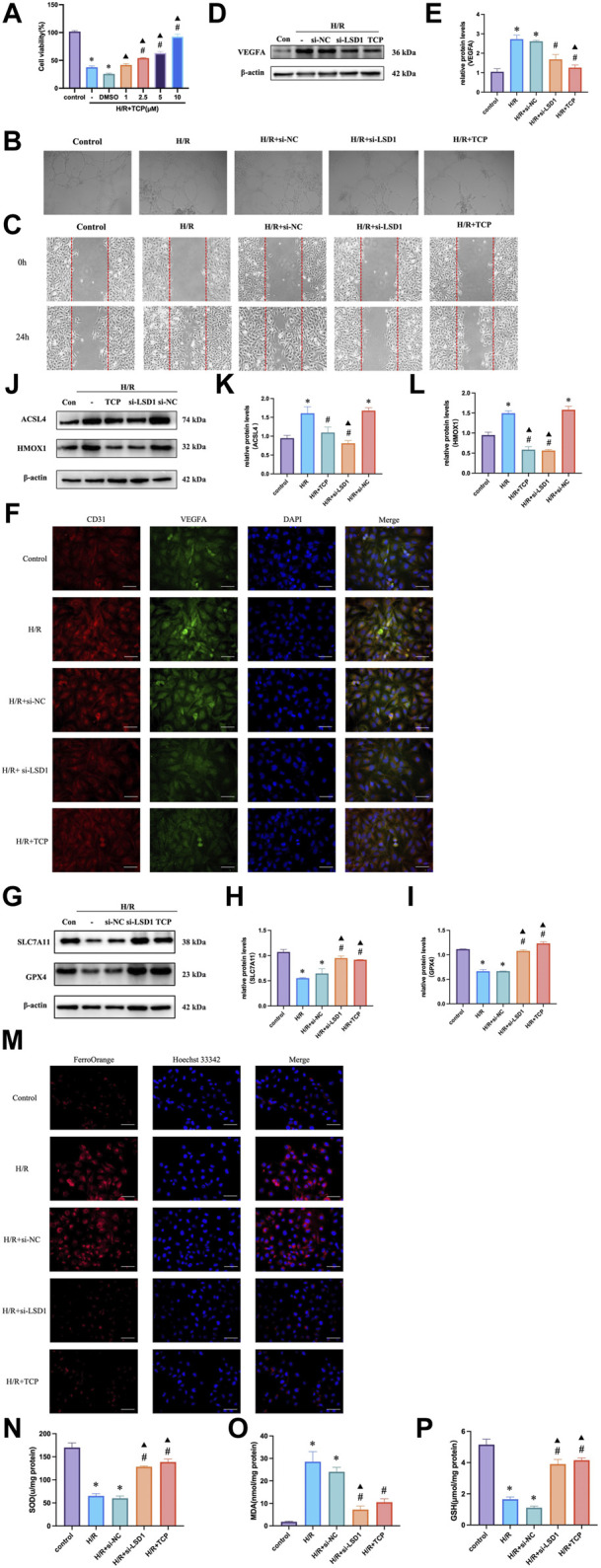
Hypoxia/reoxygenation (H/R)-induced oxidative stress and angiogenesis were decreased in human umbilical vein endothelial cells (HUVECs) by LSD1 inhibition. **(A)** Comparison of cell viability between H/R and a variety of tranylcypromine hydrochloride (TCP) treatments is shown in the CCK-8 data. **(B)** After various treatments, serum-starved HUVECs were seeded onto Matrigel and incubated (×100; scale bars = 100 μm). **(C)** The effects of TCP or si-LSD1 on the cell scratch test following H/R. **(D–E)** Western blot analysis of VEGFA expression following TCP or si-LSD1 therapy. **(F)** Double immunofluorescence staining (×400; scale bars = 50 μm) for both VEGFA and CD31 following treatment with TCP or si-LSD1. **(G–L)** ACSL4, HMOX1, GPX4, and SLC7A11 expression was quantified and compared to that in the control group. The effect of TCP or si-LSD1 on protein expression following H/R was also measured. **(M)** TCP, or si-LSD1 regulation of the Fe^2+^ concentration in HUVECs exposed to H/R (400×; scale bars = 50 μm). **(N–P)** The effect of TCP, or si-LSD1 on SOD activity, GSH levels, and MDA levels in HUVECs exposed to H/R. n = 6, the data are expressed as the mean ± SEM. For A, *p < 0.05 versus the control group, #*p* < 0.05 versus H/R group, ▲*p* < 0.05 versus H/R + DMSO group. For **(D,E, G–I, J,K, N,P)**, *p < 0.05 versus the control group, #*p* < 0.05 versus H/R group, ▲*p* < 0.05 versus H/R + si-NC group.

The Cell Counting Kit-8 (CCK-8) assay results indicated that HUVECs were more viable at a TCP concentration of 10 μM (Figure 7A). Next, we conducted tube formation and scratch assays to determine whether H/R induced angiogenic activity *in vitro* and how this was affected by TCP or si-LSD1 (Figures 7B, C). H/R-induced HUVEC migration was significantly reduced by pretreatment with an LSD1 inhibitor and by LSD1 knockdown. The WB results indicated that TCP or si-LSD1 might lower VEGFA expression (Figures 7D, E). The results of double immunofluorescence staining showed that VEGFA and CD31 expression increased concurrently following H/R and decreased following LSD1 inhibition and following si-LSD1-mediated LSD1 knockdown (Figure 7F). Our investigation revealed that following H/R treatment, the levels of ACSL4, HMOX1 (Figures 7J–L), MDA (Figure 7O), and Fe^2+^ (Figure 7M) increased, whereas those of GPX4, SLC7A11 (Figures 7G–I), SOD, and GSH (Figures 7N, P) decreased. These proteins are involved in ferroptosis and oxidative stress. Nevertheless, treatment with TCP or LSD1 knockdown reversed these alterations, suggesting that decreased angiogenesis, oxidative stress, and ferroptosis are associated with the protective effect conferred by LSD1 inhibition.

### 3.8 Inhibition of HIF-1α attenuated angiogenesis, oxidative stress and ferroptosis

**FIGURE 8:**
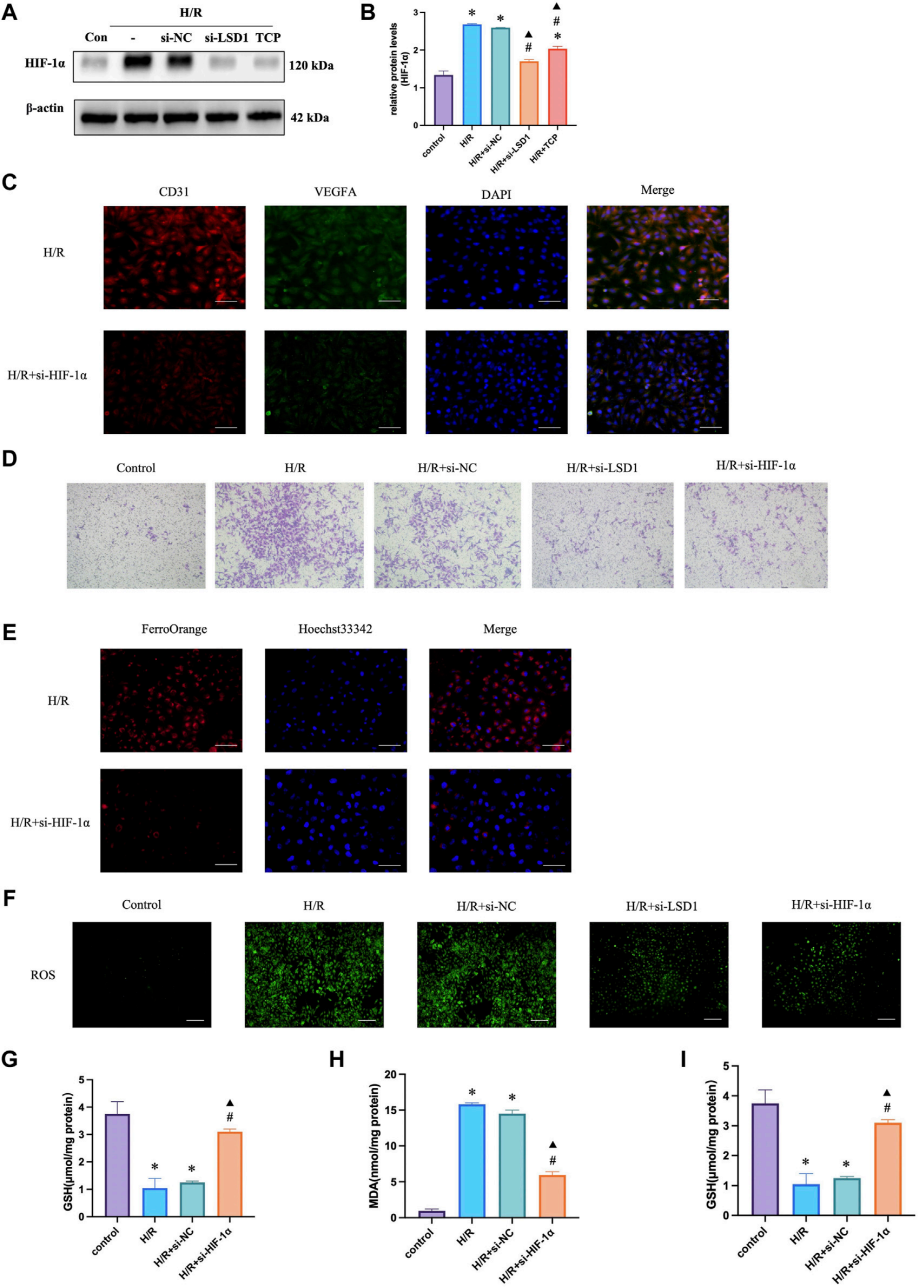
HIF-1α silencing in human umbilical vein endothelial cells (HUVECs) reduced hypoxia/reoxygenation (H/R)-induced angiogenesis, oxidative stress, and ferroptosis. Western blotting analysis of HIF-1α expression following tranylcypromine hydrochloride (TCP) treatment si-LSD1 knockdown of LSD1 expression **(A–C)**. Double immunofluorescence labeling (×400; scale bar = 50 μm) for VEGFA and CD31 following HIF-1α knockdown. **(D)** Transwell assay of HUVEC migration following si-HIF-1α transfection (×100; scale bars = 100 μm). **(E)** The effect of si-HIF-1α on the level of Fe^2+^ in HUVECs exposed to H/R (400×; scale bars = 50 μm). **(F)** Following HIF-1α silencing *in vitro*, the generation of reactive oxygen species (ROS) was quantified using 2′,7′-dichlorodihydrofluorescein diacetate molecular probes (×100; scale bars = 200 μm). **(G-I)** si-HIF-1α regulated SOD activity, GSH levels, and MDA levels in HUVECs exposed to H/R. *p < 0.05 versus the control group, #*p* < 0.05 versus H/R group, ▲*p* < 0.05 versus H/R + si-NC group.

HIF-1α is reported to be a key mediator of angiogenesis and is linked to ferroptosis and oxidative stress (Dewhirst, 2009). Therefore, we hypothesized that HIF-1α plays a role in corneal alkali burns. Our study revealed that the H/R-induced increase in HIF-1α expression was significantly reduced by TCP treatment or LSD1 knockdown (Figures 8A, B). Following HIF-1α knockdown, we observed a decrease in CD31 and VEGFA expression (Figure 8C) (400×; scale bars = 50 μm). The Transwell assay results showed that HIF-1α knockdown reduced H/R-induced HUVEC migration (Figure 8D). Moreover, Figures 8E–I shows that ROS, MDA, and Fe^2+^ levels decreased, while GSH and SOD levels increased. These findings suggest that HIF-1α regulation is crucial for controlling angiogenesis, oxidative stress, and ferroptosis.

### 3.9 LSD1 regulates angiogenesis, oxidative stress, and ferroptosis through the HIF-1α pathway

**FIGURE 9:**
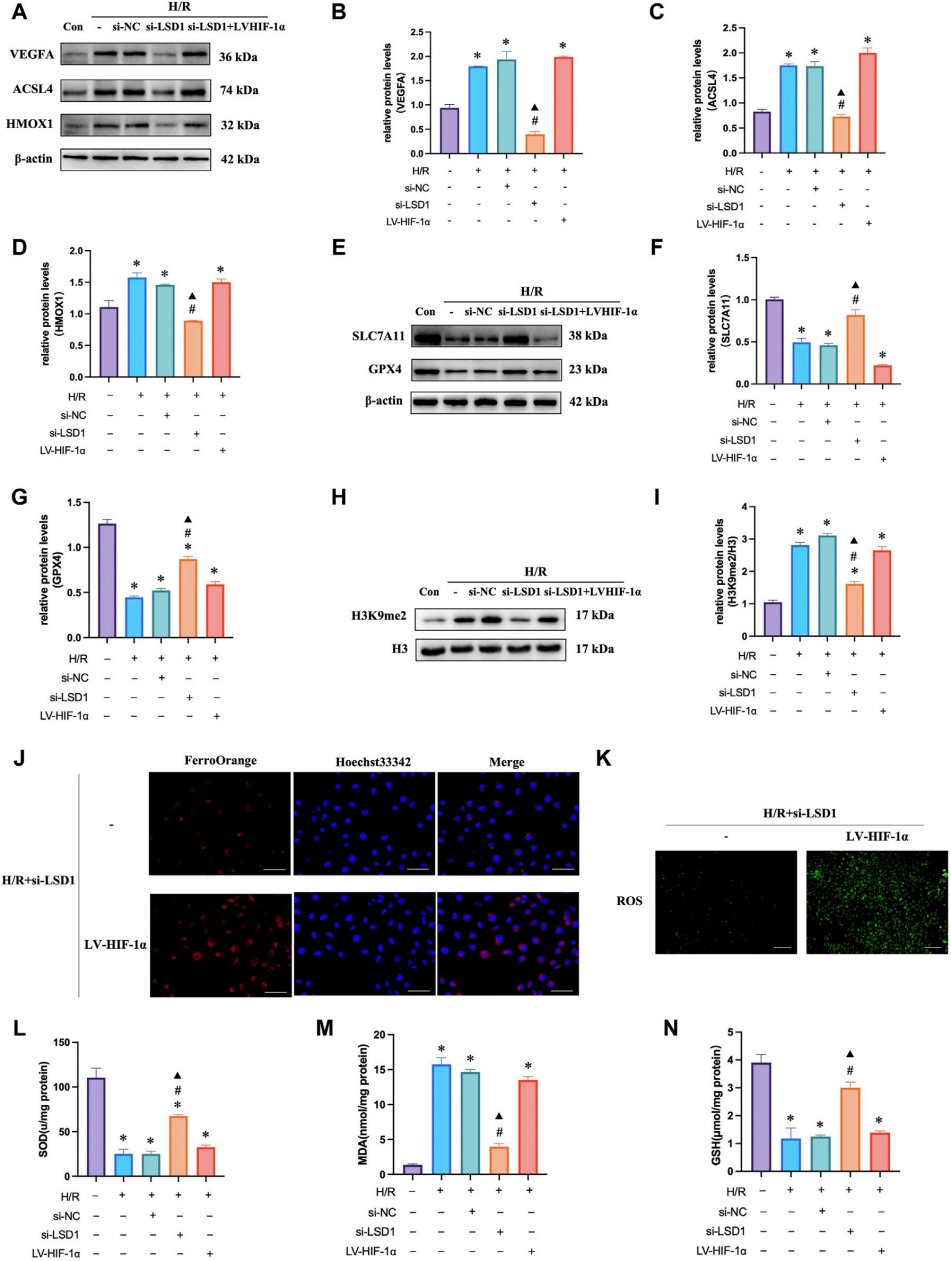
LSD1 activated the HIF-1α-associated pathway in human umbilical vein endothelial cells (HUVECs), aggravating the hypoxia/reoxygenation (H/R)-induced angiogenesis, ferroptosis, and oxidative stress. **(A–I)** Following LSD1 silencing, VEGFA, H3K9me2, ACSL4, HMOX1, GPX4, and SLC7A11 expression was analyzed by Western blotting in the presence or absence of LV-HIF-1α, and the results were quantified. **(J)** The effect of LV-HIF-1α presence or absence on Fe2+ concentration in HUVECs exposed to H/R and si-LSD1 (×400; scale bars = 50 μm). **(K)** After LSD1 silencing, the amount of reactive oxygen species (ROS) produced *in vitro* (100×; scale bars = 250 μm) with or without LV-HIF-1α was determined using 2′,7′-dichlorodihydrofluorescein diacetate molecular probes. **(L–N)** The effects of LV-HIF-1α with or without si-LSD1 on the levels of MDA, GSH, and SOD activity in HUVECs exposed to H/R. For n = 6, the data are expressed as the mean ± SEM. *p < 0.05 versus the control group, #*p* < 0.05 versus H/R group, ▲*p* < 0.05 versus H/R + si-NC group.

To investigate the mechanisms through which LSD1 regulates angiogenesis, oxidative stress, and ferroptosis, we inhibited the effects of LSD1 knockdown by transfecting HUVECs with lentiviruses overexpressing HIF-1α. The findings demonstrated that compensation by HIF-1α overexpression attenuated the observed decreases in VEGFA, H3K9me2, ACSL4, and HMOX1 expression (Figures 9A–D, H, I), as well as the decreases in Fe^2+^, MDA and ROS levels (Figures 9J, K, M). Furthermore, HIF-1α overexpression also reversed the elevated GPX4 and SLC7A11 expression levels (Figures 9E–G) and the elevated SOD and GSH levels (Figures 9L–N).

### 3.10 LSD1 suppression decreased JAK2/STAT3/HIF-1α pathway activity *in vivo*

**FIGURE 10:**
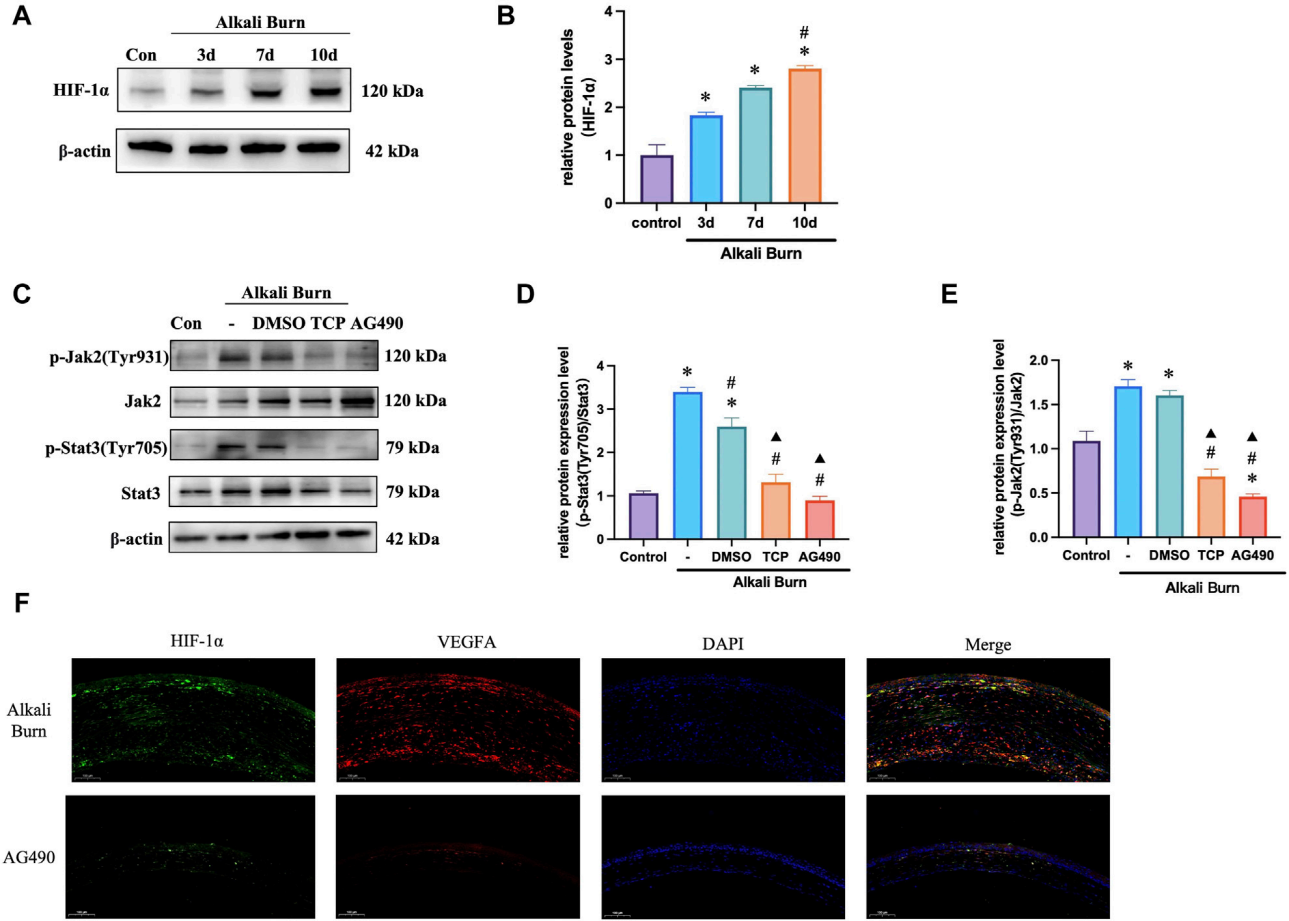
Mouse corneal alkali burn-induced JAK2/STAT3/HIF-1α activation was decreased by an LSD1 inhibitor. **(A,B)** At various postburn days, the amount of HIF-1α protein was measured, and the expression of HIF-1α was quantified and compared to that in the control group. **(C–E)** The expression of p-JAK2/JAK2 and p-STAT3/STAT3 was analyzed by Western blotting following tranylcypromine hydrochloride (TCP) or AG490 treatment, and the results were quantified. **(F)** HIF-1α and VEGFA immunofluorescence doubled in mice with corneal alkali burns treated with AG490 following. (×400; scale bars = 50 μm). n = 6, the data are expressed as the mean ± SEM. For A-B, *p < 0.05 versus the control group, #*p* < 0.05 versus 3 days group, ▲*p* < 0.05 versus 7 days group. For C-E, *p < 0.05 versus the control group, #*p* < 0.05 versus alkali burn group, ▲*p* < 0.05 versus alkali burn + DMSO group.

We next sought to validate our *in vitro* experimental results *in vivo*. The findings demonstrated that following corneal alkali burn injury, HIF-1α and LSD1 expression increased, and was markedly increased 10 days after injury (Figures10A). The JAK2/STAT3 pathway controls HIF-1α expression. Following corneal alkali burn injury, the p-JAK2/JAK2 and p-STAT3/STAT3 ratios increased and treatment with TCP partially reversed this effect. Since AG490 is a specific inhibitor of the JAK2/STAT3 pathway, we subconjunctively injected AG490 (10 mg/kg) into mice with corneal alkali burns. These observed results were comparable to those obtained for TCP (Figures 10C–F), suggesting that LSD1 may function through the JAK2/STAT3/HIF-1α pathway.

## 4 Discussion

**FIGURE 11:**
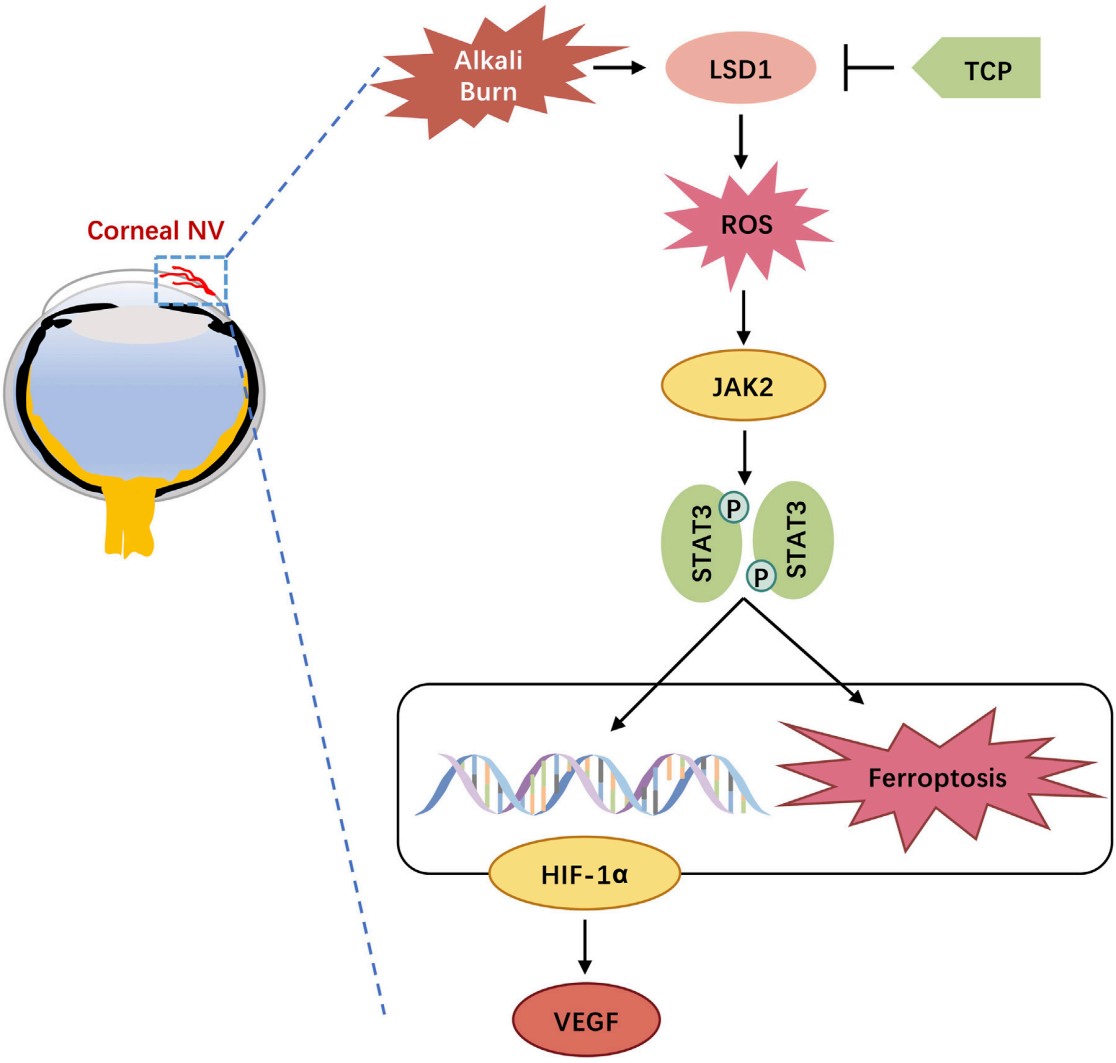
Schematic diagram of lysine-specific demethylase 1 alleviates alkali burn-induced corneal neovascularization and ferroptosis via the JAK2/STAT3/HIF-1α pathway.

In this study, we investigated the function and mechanism of action of LSD1 in a corneal neovascularization (CNV) model. We successfully used mice and HUVECs to establish CNV and H/R models. Our results demonstrated that LSD1 expression was significantly elevated following corneal alkali burn injury and that LSD1 inhibition improved corneal edema, transparency, inflammatory cell infiltration, and neovascularization. Moreover, LSD1 inhibition attenuated angiogenesis, oxidative stress, and ferroptosis induced by H/R through pathways regulated by HIF-1α. Additionally, LSD1 may regulate HIF-1α through the JAK2/STAT3 pathway (Figure 11).

Corneal alkali damage is one of the most dangerous disorders in ophthalmology. It can cause corneal opacity, inflammation, neovascularization, and abnormalities in the corneal epithelium, all of which can lead to irreversible vision loss (Sharma et al., 2018). Alkali burn-induced damage causes excessive angiogenesis and an overabundance of inflammatory responses. Our research revealed that mouse corneas are vulnerable to corneal edema and CNV, as well as extensive neovascularization 10 days after alkali burns.

VEGF is a reliable and selective endothelial cell mitogen that promotes endothelial cell growth, migration, and tube formation. VEGF is present throughout the eye and is primarily concentrated in the inner nuclear layer, retinal pigment epithelial layer, and retinal ganglion cell layer. It is also expressed in the corneal epithelium (Amadio et al., 2016). Among the VEGF family members, VEGFA is the most potent proangiogenic factor. According to Bao et al., hypoxia promotes angiogenesis and cell migration while also increasing VEGFA and interleukin-6 expression (Bao et al., 2012). Consistent with previous findings, we found that the expression of VEGFA increased significantly after corneal alkali burn injury or H/R, along with the upregulation of proangiogenic factors such as CD31. Inhibition of LSD1 attenuated these effects and downregulated the expression of inflammatory factors such as IL-6, IL-1β and TNF-α.

LSD1 is a chromatin-modifying enzyme that functions as an activator or repressor of gene expression by specifically targeting the mono- or dimethylated histones H3K4 and H3K9. LSD1 plays a role in several pathological processes, including angiogenesis, inflammation, neurogenesis, and cancer (Kim et al., 2021). Martyna Wojtala et al. reported that LSD1 increased NF-κB-dependent inflammation in human endothelial cells (Wojtala et al., 2021). Lee et al. demonstrated that LSD1 is a crucial regulator of HIF1α/VEGF-mediated tumor angiogenesis by antagonizing the crosstalk between posttranslational modifications involving HIF-1α protein degradation (Lee et al., 2017). Moreover, LSD1 siRNA ameliorated the glucose-induced reduction in H3K9me2 reduction and increase in p65 in the MMP-9 promoter region in ophthalmological diseases and prevented MMP-9 activation, mitochondrial damage, and apoptosis in diabetic retinopathy. In retinal ganglion cells, LSD1 inhibitor TCP-induced transcriptional and epigenetic regulation modulated retinal ganglion cell survival by promoting p38 MAPKγ activity. Despite this, how LSD1 controls corneal neovascularization remains unclear. In our study, the expression levels of LSD1 and H3K9me2 were considerably enhanced *in vivo* and *in vitro*. Furthermore, inhibiting LSD1 with either TCP or si-LSD1 alleviated corneal damage and inflammation following alkali burns, suggesting that LSD1 plays a critical role in controlling CNV.

Ferroptosis is a unique type of programmed cell death characterized by the excessive accumulation of lipid peroxides. It is linked to a variety of clinical events, such as liver injury, ischemia/reperfusion injury, cancer, and neurological illnesses. Notably, ferroptosis may play a role in the etiology of several ocular conditions, including photoreceptor degeneration, corneal endothelial cell abnormalities, and dysfunction of the retinal pigment epithelium (Tang et al., 2021; Zuo et al., 2022; Mi et al., 2023). Wang et al. reported that ferroptosis plays an essential role in corneal injury after alkali burns. In our investigation, ferroptosis was observed to occur in CNV. In both the CNV and H/R models, the GPX4, GSH and SLC7A11 levels decreased, whereas the ASCL4, HMOX1, 4-HNE, and Fe^2+^ levels increased. Moreover, CNV- or H/R-induced ferroptosis may be reduced through TCP or siRNA inhibition of LSD1, suggesting that LSD1 plays a role in ferroptosis induced by corneal alkali burn.

One of the primary pathogenic processes of CNV is oxidative stress, which is defined as the excessive generation of ROS or reactive nitrogen species (RNS) in response to damaging stimuli, such as UV radiation and high metabolic activity (Phaniendra et al., 2015). Notably, inflammation and ROS generation are considered drivers of ferroptosis (Sun et al., 2018). Our findings, consistent with those of earlier research, showed that corneal alkali burn-induced oxidative stress was marked by a decrease in SOD activity, an increase in MDA concentration, and elevated ROS levels following CNV or H/R. Similarly, following inhibition by TCP or si-LSD1, SOD levels increased while MDA and ROS levels decreased, suggesting that LSD1 facilitates CNV by regulating oxidative stress.

Hypoxia-inducible factor 1-alpha (HIF-1α) is a heterodimer consisting of the oxygen-regulatory subunit Hif-1α (120 kDa) and a structural subunit Hif-1β (91–94 kDa). Under hypoxia conditions, Hif-1α becomes more stable, translocates to the nucleus, and binds to Hif-1β to form the HIF-1 dimer. HIF-1 then binds to the hypoxia response element (HRE) in target genes and forms a transcription activation complex with the cofactor CBP/p300, thereby initiating target gene transcription (Semenza, 2001). A series of proangiogenic genes, such as vascular endothelial growth factor (VEGF), platelet-derived growth factor (PDGF), and angiopoietin (Ang), can be activated by HIF-1, leading to neovascularization. It has been reported that HIF-1α contributes to retinal neovascularization (Yan and Su, 2014). Futhermore, HIF-1α is a critical modulator of iron metabolism, with its upregulation resulting in decreased cellular iron accumulation (Jaakkola et al., 2001; Feng et al., 2021; Yuan et al., 2022).

In our study, alkali burn- or H/R-induced HIF-1α expression was reduced at both the mRNA and protein levels by TCP administration and LSD1 knockdown. Additionally, we delivered a lentivirus containing human HIF-1α to HUVECs to counteract the TCP-mediated decrease in HIF-1α. These findings indicate that by suppressing HIF-1α, LSD1 inhibition exerts antiangiogenic, antioxidative stress, and antiferroptotic effects. Hypoxia can affect body metabolism by activating the JAK2/STAT3 signaling pathway, which is involved in the initiation and development of inflammatory and immune responses in various pathological processes (Zhang et al., 2023). In this research, we found that the expression of phosphorylated-JAK2 (p-JAK) and phosphorylated-STAT3 (p-STAT3) decreased after LSD1 inhibition. Furthermore, the application of AG490 significantly downregulated the expression of HIF-1α and VEGFA, suggesting that LSD1 likely regulates HIF-1α through the JAK2/STAT3 pathway.

This study has several limitations. Further research should clarify the protein sites important for LSD1-mediated HIF-1α regulation elucidate protein‒protein interactions and define a more explicit mechanism of action. Moreover, the specific mechanism by which the JAK2/STAT3 pathway regulates HIF-1α needs to be further verified.

To our knowledge, this is the first work to investigate the mechanisms of interactions between LSD1 and JAK2/STAT3/HIF-1α in the field of CNV. The findings suggest that LSD1 could be a potential therapeutic target for CNV in clinical settings.

## 5 Conclusion

In sum, our findings indicate that LSD1 is a viable therapeutic target for CNV because it inhibits HIF-1α-dependent oxidative stress and ferroptosis via the JAK2/STAT3 pathway, thereby preventing corneal neovascularization. LSD1 inhibitors hold potential to be an innovative and promising treatments for CNV.

## Data Availability

The raw data supporting the conclusions of this article will be made available by the authors, without undue reservation.

## Abbreviations

CNV, corneal neovascularization; LSD1, lysine-specific demethylase 1; H3K9me1/2, histone H3 monomethyl/dimethyl lysine 9; DMEM, Dulbecco’s modified Eagle’s medium; qPCR, quantitative real-time PCR; ROS, reactive oxygen species; TCP, tranylcypromine hydrochloride; siRNA, small-interfering RNA; DMSO, Dimethyl sulfoxide; GSH, glutathione; MDA, malondialdehyde; SOD, superoxide dismutase.

## References

[B1] Amadio M. Govoni S. Pascale A. (2016). Targeting VEGF in eye neovascularization: what’s new? a comprehensive review on current therapies and oligonucleotide-based interventions under development. Pharmacol. Res. 103, 253–269. 10.1016/j.phrs.2015.11.027 26678602

[B2] Ambati B. K. Nozaki M. Singh N. Takeda A. Jani P. D. Suthar T. (2006). Corneal avascularity is due to soluble VEGF receptor-1. Nature 443, 993–997. 10.1038/nature05249 17051153 PMC2656128

[B3] Araki H. Hino S. Anan K. Kuribayashi K. Etoh K. Seko D. (2023). LSD1 defines the fiber type-selective responsiveness to environmental stress in skeletal muscle. eLife 12, e84618. 10.7554/eLife.84618 36695573 PMC9876571

[B4] Bai J.-Q. Qin H.-F. Zhao S.-H. (2016). Research on mouse model of grade II corneal alkali burn. Int. J. Ophthalmol. 9, 487–490. 10.18240/ijo.2016.04.02 27162717 PMC4853340

[B5] Bao B. Ali S. Ahmad A. Azmi A. S. Li Y. Banerjee S. (2012). Hypoxia-induced aggressiveness of pancreatic cancer cells is due to increased expression of VEGF, IL-6 and miR-21, which can be attenuated by CDF treatment. PloS One 7, e50165. 10.1371/journal.pone.0050165 23272057 PMC3521759

[B6] Bao W.-D. Pang P. Zhou X.-T. Hu F. Xiong W. Chen K. (2021). Loss of ferroportin induces memory impairment by promoting ferroptosis in Alzheimer’s disease. Cell Death Differ. 28, 1548–1562. 10.1038/s41418-020-00685-9 33398092 PMC8166828

[B7] Chang J. H. Gabison E. E. Kato T. Azar D. T. (2001). Corneal neovascularization. Curr. Opin. Ophthalmol. 12, 242–249. 10.1097/00055735-200108000-00002 11507336

[B8] Chen L. Sun B.-B. Wang T. Wang X. Li J.-Q. Wang H.-X. (2010). Cigarette smoke enhances {beta}-defensin 2 expression in rat airways via nuclear factor-{kappa}B activation. Eur. Respir. J. 36, 638–645. 10.1183/09031936.00029409 20150208

[B9] Cursiefen C. Chen L. Borges L. P. Jackson D. Cao J. Radziejewski C. (2004). VEGF-A stimulates lymphangiogenesis and hemangiogenesis in inflammatory neovascularization via macrophage recruitment. J. Clin. Invest. 113, 1040–1050. 10.1172/JCI20465 15057311 PMC379325

[B10] Das A. B. Seddon A. R. O’Connor K. M. Hampton M. B. (2021). Regulation of the epigenetic landscape by immune cell oxidants. Free Radic. Biol. Med. 170, 131–149. 10.1016/j.freeradbiomed.2020.12.453 33444713

[B11] Dewhirst M. W. (2009). Relationships between cycling hypoxia, HIF-1, angiogenesis and oxidative stress. Radiat. Res. 172, 653–665. 10.1667/RR1926.1 19929412 PMC2790140

[B12] Feng R. Xiong Y. Lei Y. Huang Q. Liu H. Zhao X. (2022). Lysine-specific demethylase 1 aggravated oxidative stress and ferroptosis induced by renal ischemia and reperfusion injury through activation of TLR4/NOX4 pathway in mice. J. Cell Mol. Med. 26, 4254–4267. 10.1111/jcmm.17444 35775122 PMC9344828

[B13] Feng X. Wang S. Sun Z. Dong H. Yu H. Huang M. (2021). Ferroptosis enhanced diabetic renal tubular injury via HIF-1α/HO-1 pathway in db/db mice. Front. Endocrinol. 12, 626390. 10.3389/fendo.2021.626390 PMC7930496 33679620

[B14] Friedmann Angeli J. P. Krysko D. V. Conrad M. (2019). Ferroptosis at the crossroads of cancer-acquired drug resistance and immune evasion. Nat. Rev. Cancer 19, 405–414. 10.1038/s41568-019-0149-1 31101865

[B15] Gao Y. Mao J. Zhang R. Deng Q. Wang Y. Pan Y. (2024). Inhibiting PRMT1 protects against CoNV by regulating macrophages through the FGF2/PI3K/Akt pathway. Eur. J. Pharmacol. 977, 176673. 10.1016/j.ejphar.2024.176673 38815785

[B16] Gu X.-J. Liu X. Chen Y.-Y. Zhao Y. Xu M. Han X.-J. (2016). Involvement of NADPH oxidases in alkali burn-induced corneal injury. Int. J. Mol. Med. 38, 75–82. 10.3892/ijmm.2016.2594 27221536 PMC4899027

[B17] Hogg S. J. Beavis P. A. Dawson M. A. Johnstone R. W. (2020). Targeting the epigenetic regulation of antitumour immunity. Nat. Rev. Drug Discov. 19, 776–800. 10.1038/s41573-020-0077-5 32929243

[B18] Hsueh Y.-J. Chen Y.-N. Tsao Y.-T. Cheng C.-M. Wu W.-C. Chen H.-C. (2022). The pathomechanism, antioxidant biomarkers, and treatment of oxidative stress-related eye diseases. Int. J. Mol. Sci. 23, 1255. 10.3390/ijms23031255 35163178 PMC8835903

[B19] Jaakkola P. Mole D. R. Tian Y. M. Wilson M. I. Gielbert J. Gaskell S. J. (2001). Targeting of HIF-alpha to the von Hippel-Lindau ubiquitylation complex by O2-regulated prolyl hydroxylation. Science 292, 468–472. 10.1126/science.1059796 11292861

[B20] Kim D. H. Im S.-T. Yoon J. Y. Kim S. Kim M. K. Chung M.-H. (2021). Comparison of therapeutic effects between topical 8-oxo-2’-deoxyguanosine and corticosteroid in ocular alkali burn model. Sci. Rep. 11, 6909. 10.1038/s41598-021-86440-7 33767351 PMC7994716

[B21] Lan F. Zaratiegui M. Villén J. Vaughn M. W. Verdel A. Huarte M. (2007). *S. pombe* LSD1 homologs regulate heterochromatin propagation and euchromatic gene transcription. Mol. Cell 26, 89–101. 10.1016/j.molcel.2007.02.023 17434129

[B22] Lee J.-Y. Park J.-H. Choi H.-J. Won H.-Y. Joo H. Shin D.-H. (2017). LSD1 demethylates HIF1α to inhibit hydroxylation and ubiquitin-mediated degradation in tumor angiogenesis. Oncogene 36, 5512–5521. 10.1038/onc.2017.158 28534506

[B23] Li J. Cao F. Yin H.-L. Huang Z.-J. Lin Z.-T. Mao N. (2020). Ferroptosis: past, present and future. Cell Death Dis. 11, 88. 10.1038/s41419-020-2298-2 32015325 PMC6997353

[B24] Linkermann A. Skouta R. Himmerkus N. Mulay S. R. Dewitz C. De Zen F. (2014). Synchronized renal tubular cell death involves ferroptosis. Proc. Natl. Acad. Sci. U. S. A. 111, 16836–16841. 10.1073/pnas.1415518111 25385600 PMC4250130

[B25] Livak K. J. Schmittgen T. D. (2001). Analysis of relative gene expression data using real-time quantitative PCR and the 2(-Delta Delta C(T)) Method. Methods San. Diego Calif. 25, 402–408. 10.1006/meth.2001.1262 11846609

[B26] Ma L. Xu A. Kang L. Cong R. Fan Z. Zhu X. (2021). LSD1-Demethylated LINC01134 confers oxaliplatin resistance through SP1-induced p62 transcription in HCC. Hepatol. Balt. Md 74, 3213–3234. 10.1002/hep.32079 34322883

[B27] Mao L. Zhao T. Song Y. Lin L. Fan X. Cui B. (2020). The emerging role of ferroptosis in non-cancer liver diseases: hype or increasing hope? Cell Death Dis. 11, 518. 10.1038/s41419-020-2732-5 32647111 PMC7347946

[B28] Mi Y. Wei C. Sun L. Liu H. Zhang J. Luo J. (2023). Melatonin inhibits ferroptosis and delays age-related cataract by regulating SIRT6/p-Nrf2/GPX4 and SIRT6/NCOA4/FTH1 pathways. Biomed. Pharmacother. Biomedecine Pharmacother. 157, 114048. 10.1016/j.biopha.2022.114048 36463827

[B29] Mu G. Zhu Y. Dong Z. Shi L. Deng Y. Li H. (2021). Calmodulin 2 facilitates angiogenesis and metastasis of gastric cancer via STAT3/HIF-1A/VEGF-A mediated macrophage polarization. Front. Oncol. 11, 727306. 10.3389/fonc.2021.727306 34604066 PMC8479158

[B30] Phaniendra A. Jestadi D. B. Periyasamy L. (2015). Free radicals: properties, sources, targets, and their implication in various diseases. Indian J. Clin. Biochem. IJCB 30, 11–26. 10.1007/s12291-014-0446-0 25646037 PMC4310837

[B31] Semenza G. L. (2001). HIF-1 and mechanisms of hypoxia sensing. Curr. Opin. Cell Biol. 13, 167–171. 10.1016/s0955-0674(00)00194-0 11248550

[B32] Sharma N. Kaur M. Agarwal T. Sangwan V. S. Vajpayee R. B. (2018). Treatment of acute ocular chemical burns. Surv. Ophthalmol. 63, 214–235. 10.1016/j.survophthal.2017.09.005 28935121

[B33] Shi Y. Lan F. Matson C. Mulligan P. Whetstine J. R. Cole P. A. (2004). Histone demethylation mediated by the nuclear amine oxidase homolog LSD1. Cell 119, 941–953. 10.1016/j.cell.2004.12.012 15620353

[B34] Sun Y. Zheng Y. Wang C. Liu Y. (2018). Glutathione depletion induces ferroptosis, autophagy, and premature cell senescence in retinal pigment epithelial cells. Cell Death Dis. 9, 753. 10.1038/s41419-018-0794-4 29988039 PMC6037763

[B35] Tan H. W. Xu Y.-M. Qin S.-H. Chen G.-F. Lau A. T. Y. (2021). Epigenetic regulation of angiogenesis in lung cancer. J. Cell Physiol. 236, 3194–3206. 10.1002/jcp.30104 33078404

[B36] Tang Z. Ju Y. Dai X. Ni N. Liu Y. Zhang D. (2021). HO-1-mediated ferroptosis as a target for protection against retinal pigment epithelium degeneration. Redox Biol. 43, 101971. 10.1016/j.redox.2021.101971 33895485 PMC8099560

[B37] Wan S. Pan Y. Yang W. Rao Z. Yang Y. (2020). Inhibition of EZH2 alleviates angiogenesis in a model of corneal neovascularization by blocking FoxO3a‐mediated oxidative stress. FASEB J. 34, 10168–10181. 10.1096/fj.201902814RRR 32562311

[B38] Wang K. Jiang L. Zhong Y. Zhang Y. Yin Q. Li S. (2022). Ferrostatin‐1‐loaded liposome for treatment of corneal alkali burn via targeting ferroptosis. Bioeng. Transl. Med. 7, e10276. 10.1002/btm2.10276 35600640 PMC9115688

[B39] Weng Q. Sun H. Fang C. Xia F. Liao H. Lee J. (2021). Catalytic activity tunable ceria nanoparticles prevent chemotherapy-induced acute kidney injury without interference with chemotherapeutics. Nat. Commun. 12, 1436. 10.1038/s41467-021-21714-2 33664241 PMC7933428

[B40] Wojtala M. Rybaczek D. Wielgus E. Sobalska-Kwapis M. Strapagiel D. Balcerczyk A. (2021). The role of lysine-specific demethylase 1 (LSD1) in shaping the endothelial inflammatory response. Cell Physiol. Biochem. Int. J. Exp. Cell Physiol. Biochem. Pharmacol. 55, 569–589. 10.33594/000000436 34612026

[B41] Yan H.-T. Su G.-F. (2014). Expression and significance of HIF-1 α and VEGF in rats with diabetic retinopathy. Asian Pac J. Trop. Med. 7, 237–240. 10.1016/S1995-7645(14)60028-6 24507647

[B42] Yu X. Niu T. Liu C. (2022). Mechanism of LSD1 in oxygen-glucose deprivation/reoxygenation-induced pyroptosis of retinal ganglion cells via the miR-21-5p/NLRP12 axis. BMC Neurosci. 23, 63. 10.1186/s12868-022-00747-3 36357913 PMC9650888

[B43] Yuan S. Wei C. Liu G. Zhang L. Li J. Li L. (2022). Sorafenib attenuates liver fibrosis by triggering hepatic stellate cell ferroptosis via HIF-1α/SLC7A11 pathway. Cell Prolif. 55, e13158. 10.1111/cpr.13158 34811833 PMC8780895

[B44] Zhang F. Cao X. Zhao C. Chen L. Chen X. (2023). Empagliflozin activates JAK2/STAT3 signaling and protects cardiomyocytes from hypoxia/reoxygenation injury under high glucose conditions. J. Thromb. Thrombolysis 55, 116–125. 10.1007/s11239-022-02719-0 36396837

[B45] Zhao D. Zhao Y. Wang J. Wu L. Liu Y. Zhao S. (1979). Long noncoding RNA Hotair facilitates retinal endothelial cell dysfunction in diabetic retinopathy. Clin. Sci. Lond Engl. 134, 2419–2434. 10.1042/CS20200694 32812634

[B46] Zhao M. Wang S. Zuo A. Zhang J. Wen W. Jiang W. (2021). HIF-1α/JMJD1A signaling regulates inflammation and oxidative stress following hyperglycemia and hypoxia-induced vascular cell injury. Cell Mol. Biol. Lett. 26, 40. 10.1186/s11658-021-00283-8 34479471 PMC8414688

[B47] Zuo X. Zeng H. Wang B. Yang X. He D. Wang L. (2022). AKR1C1 protects corneal epithelial cells against oxidative stress-mediated ferroptosis in dry eye. Invest. Ophthalmol. Vis. Sci. 63, 3. 10.1167/iovs.63.10.3 PMC9463717 36066316

