# Supplementary material for: LSD1 inhibition by tranylcypromine hydrochloride reduces alkali burn-induced corneal neovascularization and ferroptosis by suppressing HIF-1α pathway
Source: Front Pharmacol. 2024 Jul 26;15:1411513. doi: 10.3389/fphar.2024.1411513 (PMC11316257; doi:10.3389/fphar.2024.1411513)
Supplement: Supplementary file 1 [file Table1.DOCX]

Supplementary Material

# Main reagents and their corresponding brand numbers utilized in this paper

| Reagents | Brands | Reference |
| --- | --- | --- |
| Tranylcypromine hydrochloride (TCP) | MedChemExpress | HY-17447A |
| AG490(Tyrphostin AG490) | MedChemExpress | HY-12000 |
| Dimethyl sulfoxide (DMSO) | MedChemExpress | HY-Y0320 |
| RNA Isolater Total RNA Extraction Reagent | Vazyme | R401-01 |
| HiScript II Q RT SuperMix for qPCR | Vazyme | R222-01 |
| 2X Universal SYBR Green Fast qPCR Mix | ABclonal | RK21203 |
| Anti-LSD1 primary antibody | Abcam | ab17721 |
| Anti-CD31 primary antibody | Invitrogen | PA5-32321 |
| Anti-p-STAT3 primary antibody | Cell Signaling Technology | #9145 |
| Anti-STAT3 primary antibody | Cell Signaling Technology | #4904 |
| Anti-H3 primary antibody | Cell Signaling Technology | #4499 |
| Anti-β-actin primary antibody | Proteintech | 66009-1-Ig |
| Anti-VEGFA primary antibody | Proteintech | 19003-1-AP |
| Anti-GPX4 primary antibody | Proteintech | 67763-1-Ig |
| Anti-HMOX1 primary antibody | Proteintech | 10701-1-AP |
| Anti-ACSL4 primary antibody | Proteintech | 22401-1-AP |
| Anti-SLC7A11 primary antibody | Proteintech | 26864-1-AP |
| Anti-JAK2 primary antibody | Affinity | AF6022 |
| Anti-p-JAK2(Tyr931) primary antibody | Affinity | AF3024 |
| Anti-HIF-1α primary antibody | Affinity | AF1009 |
| Anti-H3K9me2 primary antibody | Affinity | DF6937 |
